# Topoisomerase II deficiency leads to a postreplicative structural shift in all *Saccharomyces cerevisiae* chromosomes

**DOI:** 10.1038/s41598-021-93875-5

**Published:** 2021-07-22

**Authors:** Jessel Ayra-Plasencia, Cristina Ramos-Pérez, Silvia Santana-Sosa, Oliver Quevedo, Sara Medina-Suárez, Emiliano Matos-Perdomo, Marcos Zamora-Dorta, Grant W. Brown, Michael Lisby, Félix Machín

**Affiliations:** 1 Unidad de Investigación, Hospital Universitario Nuestra Señora de la Candelaria, Ctra del Rosario 145, 38010 Santa Cruz de Tenerife, Spain; 2 Escuela de Doctorado y Estudios de Postgrado, Universidad de La Laguna, Santa Cruz de Tenerife, Spain; 3 Department of Biochemistry and Donnelly Centre, University of Toronto, Toronto, Canada; 4 Department of Biology, University of Copenhagen, Copenhagen, Denmark; 5 Instituto de Tecnologías Biomédicas, Universidad de la Laguna, Santa Cruz de Tenerife, Spain; 6 Facultad de Ciencias de la Salud, Universidad Fernando Pessoa Canarias, Las Palmas de Gran Canaria, Spain; 7 Present Address: Centro Atlántico del Medicamento (CEAMED) S.A., Santa Cruz de Tenerife, Spain; 8 Present Address: BenchSci Analytics Inc., Toronto, Canada; 9 Present Address: River Stone Biotech, Copenhagen, Denmark

**Keywords:** Cell division, Chromosomes

## Abstract

The key role of Topoisomerase II (Top2) is the removal of topological intertwines between sister chromatids. In yeast, inactivation of Top2 brings about distinct cell cycle responses. In the case of the conditional *top2-5* allele, interphase and mitosis progress on schedule but cells suffer from a chromosome segregation catastrophe. We here show that *top2-5* chromosomes fail to enter a Pulsed-Field Gel Electrophoresis (PFGE) in the first cell cycle, a behavior traditionally linked to the presence of replication and recombination intermediates. We distinguished two classes of affected chromosomes: the rDNA-bearing chromosome XII, which fails to enter a PFGE at the beginning of S-phase, and all the other chromosomes, which fail at a postreplicative stage. In synchronously cycling cells, this late PFGE retention is observed in anaphase; however, we demonstrate that this behavior is independent of cytokinesis, stabilization of anaphase bridges, spindle pulling forces and, probably, anaphase onset. Strikingly, once the PFGE retention has occurred it becomes refractory to Top2 re-activation. DNA combing, two-dimensional electrophoresis, genetic analyses, and GFP-tagged DNA damage markers suggest that neither recombination intermediates nor unfinished replication account for the postreplicative PFGE shift, which is further supported by the fact that the shift does not trigger the G_2_/M checkpoint. We propose that the absence of Top2 activity leads to a general chromosome structural/topological change in mitosis.

## Introduction

Among the physical impediments that preclude sister chromatid segregation in anaphase, there are topological intertwinings (catenanes), unfinished replication and unresolved recombination intermediates. The presence of any of these structures gives rise to anaphase bridges that can seriously compromise the genome integrity of the immediate cell lineage^1–3^. Surprisingly, cells from most organisms apparently lack specialised checkpoints to detect these aberrant structures and stop anaphase onset. Rather, they rely on indirect ways to supervise putative segregation problems ahead. For instance, during DNA replication, cells check that the replication fork (RF) does not get stalled or blocked, or that long stretches of single-stranded DNA (ssDNA) are not left behind the RF, yet cells can enter anaphase with unfinished replication if it proceeds too slowly compared to the cell division rate^1,4,5^. Likewise, cells monitor both DNA double-strand breaks (DSBs) and ssDNA during DNA damage and early steps of its repair through the homologous recombination (HR) pathway, but not the direct presence of recombination intermediates that connect the damaged DNA with its sister template^6–8^. Catenations also appear invisible to cell cycle checkpoints, although there still exist controversy about putative G_2_/M checkpoint(s) that sense these topological problems in higher eukaryotes^1,9–11^.

Eukaryotic type II topoisomerases (topo II/Top2) are exclusive in removing double-strand DNA (dsDNA) catenations^10,12^. The yeast *Saccharomyces cerevisiae* has been extensively used as a model organism where to assess the physiological roles of Top2. This has been facilitated by the simplicity of this cell model and its genetic engineering, the presence of just one *TOP2* gene, and the ability to generate simple conditional alleles; e.g., *top2* thermosensitive (ts) alleles. Pioneering work in David Botstein’s and Rolf Sternglanz’s labs showed that *top2-ts* yeast cells died as a consequence of passing through anaphase^13,14^. Later work demonstrated that Top2 was needed to avoid anaphase bridges, and that completion of cytokinesis had a major role in killing the cell progeny as it severs these anaphase bridges^15–17^. While the presence of anaphase bridges comprising sister chromatid intertwinings is undisputed, several works have suggested the presence of other linkages that could contribute to the sister chromatid segregation defects in *top2-ts*. Thus, unfinished replication has been observed and mapped to chromosome fragile sites, likely coincident with replication termini sites^18^. Replication defects have also been seen at the ribosomal DNA array (rDNA), especially when Top2 deficiency is combined with other mutations that affect rDNA metabolism^19^. The rDNA locus, located on the chromosome XII right arm, is known to be unique because of its unidirectional replication mechanism, the presence of genetically-programmed RF blocks (RFBs), being highly transcribed by RNA polymerases I and III while mostly epigenetically silent for RNA polymerase II, and for being hyper-recombinogenic and, consequently, expected to present more recombination intermediates than other chromosome regions^2,20^.

Confounding matters, not all *top2-ts* alleles bring about the same phenotypes. Whereas most of them (but not all) allow a timely anaphase onset, progression beyond this point is more variable, particularly the degree of cytokinetic completion^14,15,21,22^. The underlying reasons behind these differences are somewhat elusive, although features such as residual Top2-ts activity, the genetic background, and the capability to activate a checkpoint that transiently blocks cytokinesis (NoCut/Abscission checkpoint) might be responsible^22^. In previous works we showed that *top2-5* cells were excellent in both synchronously entering anaphase and quickly severing anaphase bridges through cytokinetic furrow ingression^15,23^. In this report, we have studied in more detail the *top2-5* cell cycle and found that all chromosomes fail separation by pulsed-field gel electrophoresis (PFGE). Except for the rDNA-bearing chromosome XII, chromosomes stop entering the PFG not at early S-phase but in mitosis. The use of mutants and mitotic drugs showed that the PFGE behaviour is independent of HR, spindle forces and, probably, anaphase onset. We propose that Top2 deficiency (*top2-5*) gives rise to substantial chromosome topological and/or structural changes that cannot however trigger an efficient G_2_/M DNA damage checkpoint. Finally, we show that Top2 actions must take place at the correct time, after which the change in chromosome structure becomes refractory to Top2 activity.

## Results

### Yeast chromosomes stop entering a pulsed-field electrophoretic gel in *top2-5*

In a previous work, we used genetically-modified *TOP2* and *top2-5* ts strains to analyse their first cell cycle by both population and single-cell fluorescence microscopy^15^. Specifically, we GFP-tagged the histone H2A2 (*HTA2* gene) in *bar1* derivatives of the original strains in order to synchronously follow the nuclear cell cycle. We started the current work by adding flow cytometry (FACS) and Pulsed Field Gel Electrophoresis (PFGE) to the microscopic analysis. Whereas fluorescence microscopy assesses cell and nuclear morphology as markers of cell cycle progression and nuclear segregation, FACS allows determination of both bulk DNA replication and of the degree of uneven segregation after cytokinesis. In addition, PFGE, in conjunction with Southern blot analysis, gives insights into the structural integrity of individual chromosomes; i.e., intact vs broken chromosomes, gross chromosomal rearrangements, and presence of DNA-mediated linkages^7,24–26^. In the case of the latter scenario, affected chromosomes are trapped in the wells of the PFG.

**Figure 1:**
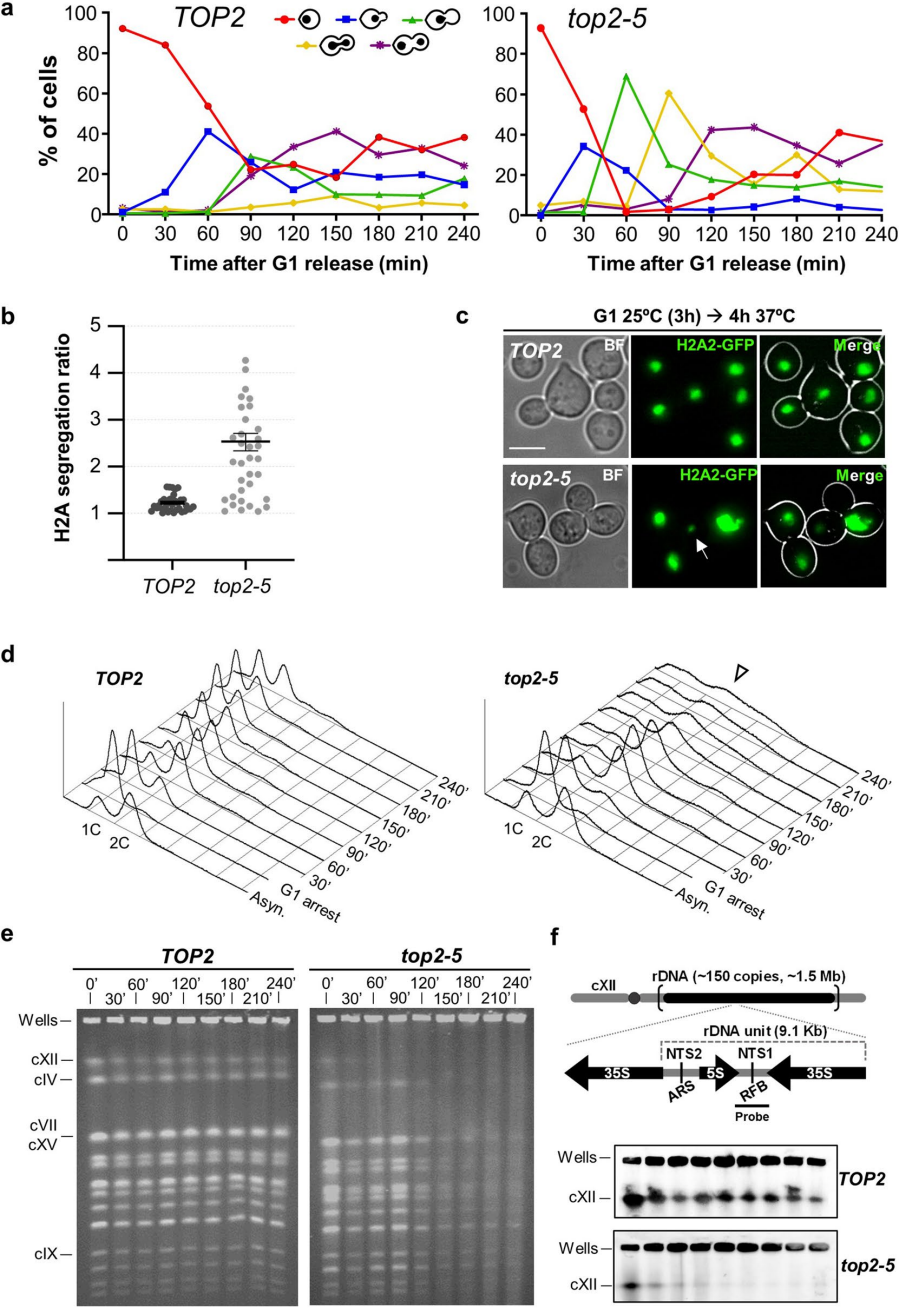
Chromosome integrity is compromised upon inactivation of Top2 with the *top2-5* thermosensitive allele. A synchronous G_1_ release experiment was performed for isogenic *TOP2* and *top2-5* strains. (**a**) Charts depicting the cell cycle progression under the microscope (both strains carry H2A2-GFP to label the nuclear masses). (**b**) Ratio of H2A2-GFP segregation among binucleated cells (n = 34; 210′–240′ after the G1 release). The ratio is calculated by dividing the upper by the lower GFP signal in each pair of nuclei. (**c**) Micrographs of representative cells 4 h after the G1 release. The arrow points to a massive uneven segregation of the H2A2-GFP signal in *top2-5*. (**d**) Flow cytometry (FACS) analysis of the DNA content. Arrowhead highlights the flattened DNA content observed in *top2-5* at later time points. (**e**) Ethidium bromide (EtBr) staining of whole chromosomes resolved by Pulsed-Field Gel Electrophoresis (PFGE). Note how in the *top2-5* strain the cXII in-gel signal disappears shortly after the G_1_ release, whereas the other chromosome bands get fainter after 120′. (**f**) Southern blot profiles of the same PFGEs with a probe against the non-transcribed spacer region 1 (NTS1) of the ribosomal DNA array (rDNA) on the cXII (schematic on the top; uncropped blots in Figure S4a).

Both strains were arrested in G_1_ at permissive temperature (25 °C) for 3 h before being released to 37 °C for 4 h. Every 30′, samples were collected for analysis by microscopy, FACS and PFGE. As reported before, fluorescence microscopy showed that the *TOP2* strain proceeded into a normal cell cycle (Fig. 1a, left panel); S-phase entry (budding) occurred between 30′ and 90′ and anaphase onset (H2A2-GFP segregation) between 90′ and 150′. Shortly after anaphase entry, cells completed the first cell cycle and split mother and daughter cells (new rise of unbudded category by 180′). We observed an initially similar cell cycle profile in the case of *top2-5* (Fig. 1a, right panel), yet with an earlier G_1_-S transition^15^; S-phase entry at 30′–60′ and the peak of anaphase onset at 90′. As reported in our previous work, we seldom observed genuine H2A2-GFP anaphase bridges in *TOP2* (< 10%) and they were transient in *top2-5* (~ 60% of cells at 90′). Instead, binucleated cells was the major segregation phenotype by 120′, indicative of cytokinetic furrow ingression by that time point^15^. This phenotype may encompass either cells in anaphase/telophase or mother and daughter cells in the next G_1_ before completing physical separation^23^. Strikingly, and unlike *TOP2*, a clear uneven segregation of the H2A2-GFP was evident in *top2-5* (Fig. 1b,c). *TOP2* and *top2-5* also differed in that ~ 20% *top2-5* mother cells rebudded without separation of the first mother and daughter (Fig. 1c)^15,23^.

FACS analysis confirmed that both strains have a timely cell cycle, at least until anaphase onset (Fig. 1d); S-phase entry (drop of 1C, rise of 2C DNA content) at 30′–60′. Cytokinesis and cell separation took place at 120′ in *TOP2* (new rise of 1C peak). Likewise, *top2-5* executed cytokinesis at 150′–180′ (drop of 2C peak); however, the outcome of such point-of-no-return was catastrophic. The 2C peak did not revert to the typical 1C DNA content but, instead, extended from < 1C to > 2C DNA content, indicating massive uneven segregation of the genetic material, as suggested by the H2A2-GFP microscopy.

When we performed the PFGE analysis, we observed that the chromosome staining pattern in *TOP2* was constant throughout the time course (Fig. 1e, left). By contrast, there were two waves of decreased chromosome staining in *top2-5* (Fig. 1e, right; Figure S1). In the first wave, the signal for the largest chromosome, chromosome XII (cXII), decreased coincident with the cells transiting through S-phase. The second wave occurred after the nuclear division but before the decrease of the 2C peak by FACS (t = 120′–240′), and was marked by a sharp and profound decrease of all chromosome staining (the in-gel signal dropped ~ 90% in less than 1 h; Figure S1). We found that decrease in chromosome staining in *top2-5* persisted through different sample preparation conditions (Figure S2). Furthermore, the decrease in chromosome staining was not observed if cells were maintained in G_1_ through an equivalent incubation regime (6 h from the induction of the G_1_ block) (Figure S3).

The lack of visible chromosomes in a PFGE can be due to three major causes: chromosome breakage, chromosome degradation and chromosomes with branched structures that keep them trapped into the loading well^7,24,25^. In order to distinguish between these possibilities, we did Southern blots under different stringency conditions with probes for the rDNA in cXII (Figs. 1f and S4). We observed strong hybridization signals in the wells for both strains. No signs of broken chromosomes (fast-migrating smear, e.g.) were noted (Figure S4). We did not observe signs of DNA degradation over the 4 h time course in the *top2-5* mutant, even after the massive missegregation of the genetic material (Figure S5). These results suggest that chromosomes get trapped in the loading well in the *top2-5* mutant.

We previously compared the *top2-5* allele with the broadly-used *top2-4* allele^15^. We found that *top2-5* transits through anaphase faster and more synchronously than *top2-4*, so that *top2-5* appears to be a better allele for cell cycle studies at this late stage. However, in order to address if our observations were specific to the *top2-5* allele, we checked the PFGE pattern in an isogenic *top2-4* strain. We found the same steady disappearance of all yeast chromosomes, yet to a lesser extent (Figure S6).

Overall, we conclude that Top2 inactivation brings about an unreported shift in the behaviour of all yeast chromosomes in a PFGE. In a synchronous cell cycle, this shift takes place in late anaphase, near or after cytokinesis.

### Cytokinesis is not responsible for the *top2-5* structural chromosome change revealed by PFGE

**Figure 2:**
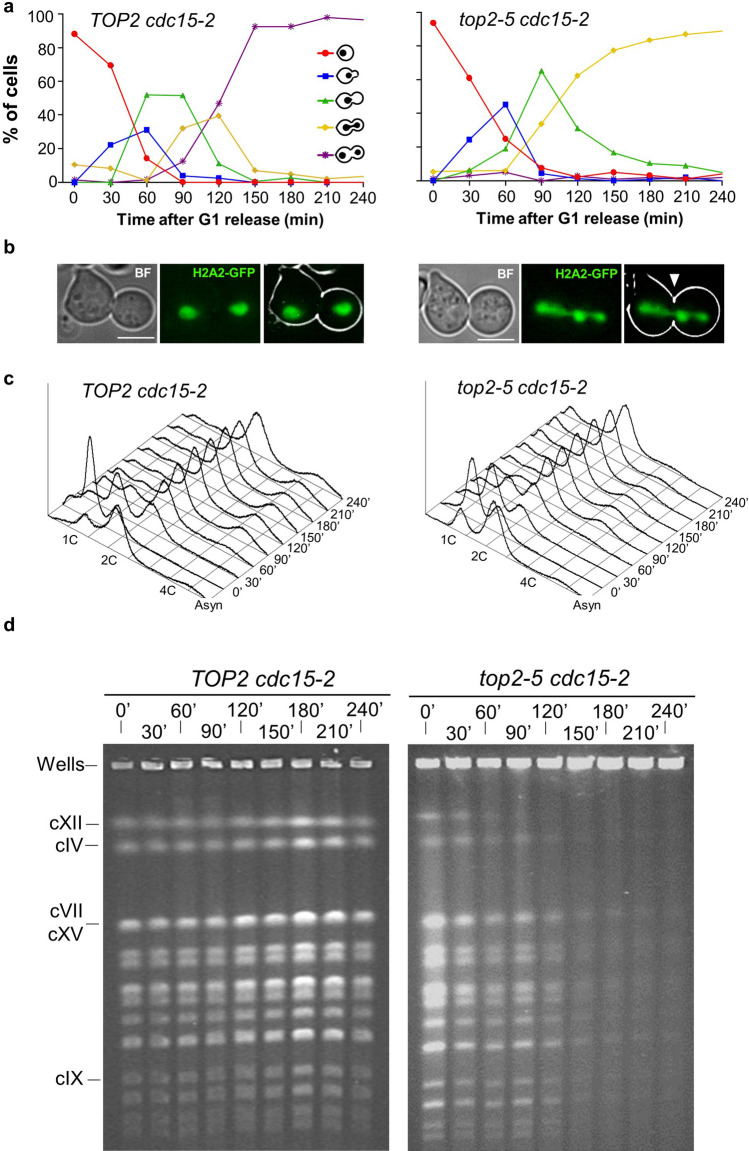
The loss of chromosome integrity in *top2-5* is not a consequence of the mitotic catastrophe that happens upon cytokinesis. A synchronous G_1_ release experiment was performed for isogenic *TOP2 cdc15-*2 (left panels) and *top2-5 cdc15-*2 (right panels) strains. (**a**) Charts depicting the cell cycle progression under the microscope. (**b**) Micrographs of representative cells 4 h after the G_1_ release. The arrowhead points to the characteristic massive H2A2-GFP anaphase bridge observed in *top2-5 cdc15-2*. (**c**) FACS analysis of the DNA content. (**d**) EtBr staining of whole chromosomes resolved by PFGE. Note how chromosome behaviour of the *top2-5 cdc15-2* strain resembled that of *top2-5*.

The general loss of chromosome bands in the *top2-5* PFGE from 120′ onwards could simply be due to cells completing a devasting cytokinesis, as both microscopy and FACS strongly suggest. Cytokinesis would break chromosomes at the anaphase bridges; and these broken chromosomes could get trapped in the PFGE well during DSB end resection^25^. Of note, we and others have shown that cytokinesis is a point of no return during Top2 inactivation^15,17^. In order to test this hypothesis, we used a *top2-5 cdc15-2* double ts mutant that blocks cytokinesis and cell progression beyond telophase because of the lack of the Mitotic Exit Network (MEN) kinase Cdc15^2,15^. We repeated the time course and analysed samples by fluorescence microscopy, FACS and PFGE, comparing *top2-5 cdc15-2* with its *TOP2 cdc15-2* counterpart (Fig. 2). As expected, cells from both strains arrested in telophase, as can be seen by microscopy (dumbbells prevailed) and flow cytometry (2C peak prevailed) (Fig. 2a–c). Incidentally, a minor 4C peak appeared during the time course in both *cdc15-2* strains. This peak correlated to a 2C peak at the G_1_ arrest, which stems from incomplete septations at permissive conditions (Figure S7). As previously reported^15^, *top2-5 cdc15-2* formed histone-labelled anaphase bridges (up to 90% of cells by 210′), whereas two equally segregated histone masses was the major outcome in the *TOP2 cdc15-2* (Fig. 2a,b). Strikingly though, the PFGE of the *top2-5 cdc15-2* showed the same pattern of decrease in chromosome band intensity (Fig. 2d) as we had observed in *top2-5 CDC15.* Specifically, (i) in-gel cXII quickly disappeared (60′–90′), and (ii) all other chromosomes bands decreased at 120′–150′. We therefore conclude that the structural chromosome changes revealed by PFGE are not simply a consequence of the breakage of *top2-5* anaphase bridges by cytokinesis.

### Assessment of unfinished replication in *top2-5*

**Figure 3:**
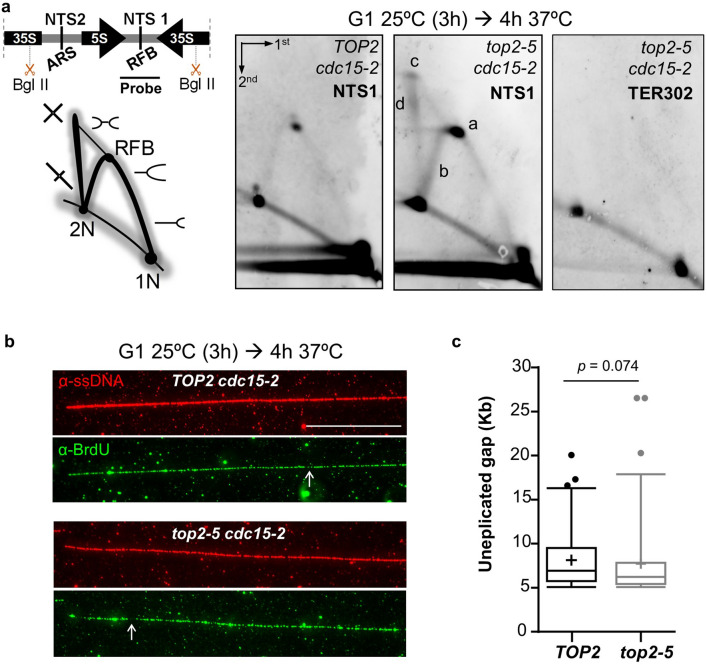
Assessment of the presence of underreplicated chromosomes in *top2-5*. Synchronous G_1_ release experiments were performed for isogenic *TOP2 cdc15-*2 and and *top2-5 cdc15-*2 strains. (**a**) The presence of persistent replication and recombination-like intermediates were evaluated by neutral, neutral two-dimensional electrophoresis (NN-2D) for the NTS1 region of the 9.1 kbp unit of the rDNA and the replication termini site *TER302*. On the left, schematic of the rDNA unit with its main features and relative position of the BglII sites and the probe; a representation of the theoretical branched forms visualized by NN-2D is depicted underneath. NTS1 and 2, non-transcribed regions 1 and 2, respectively; ARS, autonomous replicating sequence; RFB, replication fork block. On the right, NN-2D of both strains 4 h after the G_1_ release into 37 ºC. Note that four structures were enriched at NTS1 in *top2-5 cdc15-2* relative to *TOP2 cdc15-2*: the Y-shaped intermediate stalled at the RFB (“a”), the long Y-shaped intermediates that pass such block (“b”), the intermediate with highest mass and symmetry (“c” spot, probably a double Y), and the X-shaped spike (“d”). (**b**) Combing analysis of the completion of replication for DNA fibers obtained 4 h after the G1 release. Representative pictures of the 4 h immunofluorescence against DNA (red) and incorporated BrdU (green). White arrows point to the small greenless gaps observed in fibers coming from both strains. The scale bar represents 50 kbp. (**c**) Quantification of the length of gaps greater than 5 kbp.

Since cytokinesis is not required for the loss of chromosome bands in PFGE, we tested whether long-lasting DNA-DNA sister chromatid junctions are responsible^7,24,25^. The timing of the 2C peak by FACS suggests that there is no a major delay in ongoing replication; however, certain late replication intermediates in *top2-5* might change the chromosome structure in such a way that chromosomes cannot enter the PFGE. Indeed, previous results showed that yeast cells deficient in Top2 struggle to complete replication and accumulate late replication intermediates at replication termini sites^18^. Thus, we tested whether *top2-5 cdc15-2* accumulates replication intermediates well into the late anaphase block by performing neutral–neutral two-dimensional electrophoresis (NN-2D). This technique can detect the presence of branched DNA structures and classify them into replication-like (Y-shaped) and recombination-like (X-shaped) intermediates^27^. We studied two replication termini; the well-defined RFB in the rDNA, and a RF converging locus in chromosome III (*TER302*)^18^. We found enrichment of Y shapes near the RFB in *top2-5 cdc15-2* (Fig. 3a). The fixed Y-shaped structure at the RFB (spot “a” in Fig. 3a) and other longer Ys that bypass the RFB into the adjacent 3′ end of the 35S gene (“b” in Fig. 3a) were particularly enriched. In addition, other spots onto the X-shaped structures also accumulated (“c” and “d” at the spike in Fig. 3a), which might correspond to two very close converging Ys as they cannot branch-migrate along the spike. Conversely, we could not detect unfinished replication at *TER302* (Fig. 3a, rightmost blot).

Even though we could not detect late replication intermediates at the *TER302* locus, we must state that, for chromosomes other than XII, it is difficult to determine where two replication forks converge, which is expected to be variable in the cell population. Thus, we also opted for an alternative technical approach based on measuring underreplicated gaps on extended DNA fibers. These gaps would suggest the presence of two converging RFs (double Ys) in a single-molecule analysis. For that purpose, we transferred the *top2-5* and *cdc15-2* alleles to a strain suitable for the DNA combing technique^28^. We could not see differences between the *top2-5 cdc15-2* and *TOP2 cdc15-2* strain in late anaphase (Fig. 3b,c). Fibers appeared almost fully replicated in both cases; i.e., green (replicated DNA) and red (DNA) signals extensively overlapped. Although there were some gaps in the BrdU signal on the DNA fibers, there were no differences between the strains in terms of underreplicated percentage (5.85% and 5.34% for *TOP2* and *top2-5*, respectively) and track length (Fig. 3c). The number of gaps (greater than 5 kbp) was also equivalent in the two strains (72 & 69 gaps in 10 Mbp for *TOP2* and *top2-5*, respectively). The data suggest that unreplicated regions larger than 5 kbp do not accumulate in *top2-5*, although Y-structures at the rDNA RFB and X-structures at the rDNA do accumulate.

### Assessment of unresolved recombination intermediates in *top2-5*

**Figure 4:**
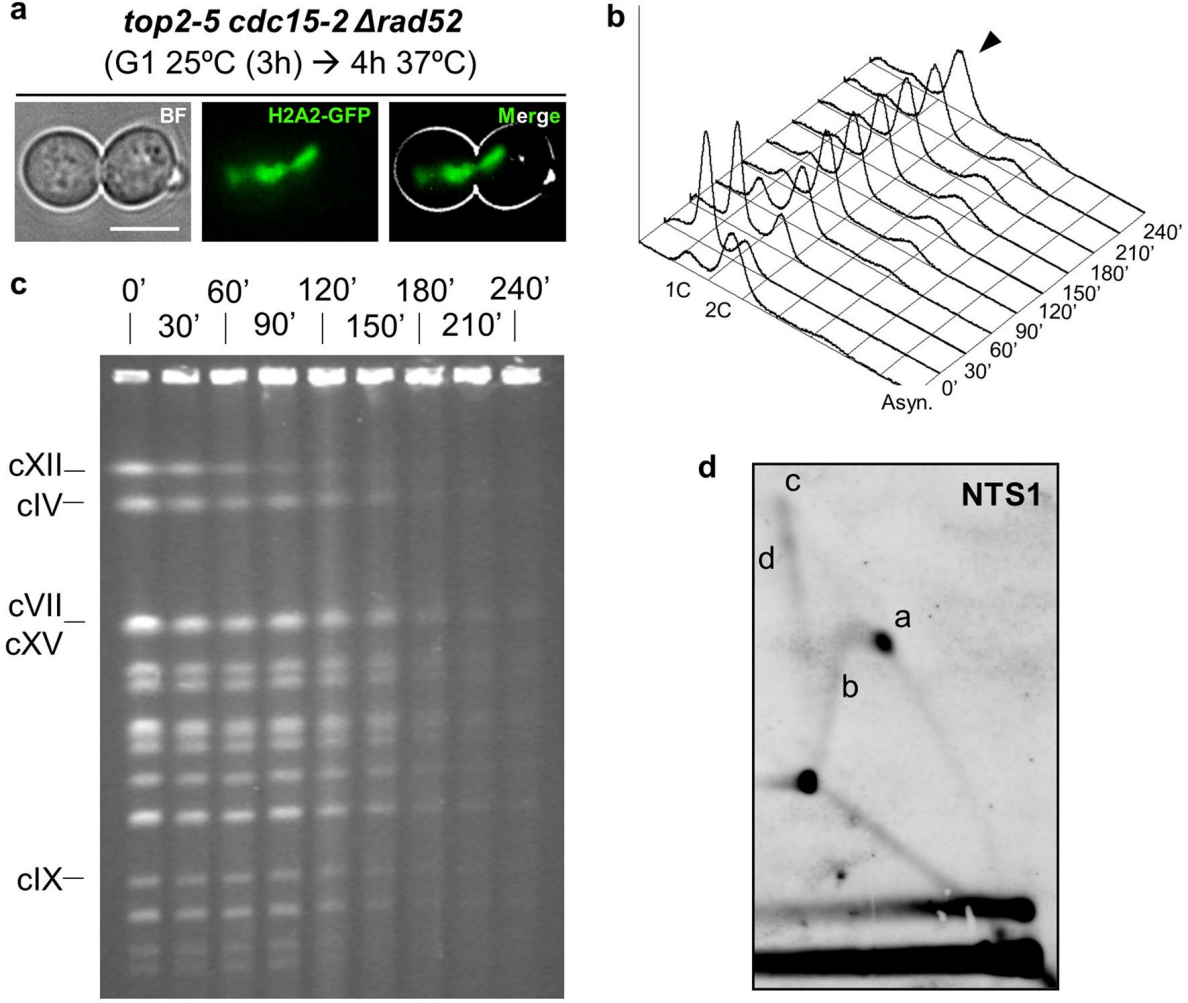
Chromosome integrity loss in *top2-5* is independent of the homologous recombination pathway. A synchronous G_1_ release experiment was performed for the *top2-5 cdc15-*2 Δ*rad52* strain. (**a**) Micrographs of a representative cell 4 h after the G_1_ release. Note it resembles *top2-5 cdc15-*2 with its characteristic H2A2-GFP anaphase bridge. (**b**) FACS analysis of the DNA content. (**c**) EtBr staining of whole chromosomes resolved by PFGE. Note how the late chromosome shift behaviour resembled that of the *top2-5 cdc15-2* strain. (**d**) NN-2D of the NTS1 region of the rDNA 4 h after the G_1_ release.

The X-shaped molecules observed by NN-2D in the *top2-5* rDNA suggest that recombination intermediates might also contribute to the trapping of chromosomes in the PFGE well. HR is known to bypass stalled RFs and might help in completing replication in *top2* mutants^29^. In order to assess the contribution of recombination intermediates to the *top2-5* PFGE and NN-2D profiles, we used a genetic approach since such intermediates depend on the HR gene *RAD52*^30^. The triple mutant *top2-5 cdc15-2 rad52*Δ strain showed a similar cell cycle profile than *top2-5 cdc15-2*, including anaphase bridges as the end-point phenotype in telophase (Fig. 4a), similar replication timing by FACS (Fig. 4b), and similar kinetics for PFGE chromosome entrapment (Fig. 4c). Thus, the abrogation of Rad52 did not prevent the late loss of chromosome bands in the PFGE. Surprisingly, it did not affect the presence of X-shaped intermediates at the rDNA either (Fig. 4d). We conclude that recombination intermediates are unlikely to contribute to the *top2-5* anaphase decrease in chromosome bands by PFGE.

### The *top2-5* structural chromosome change revealed by PFGE is independent of *CDC14*-mediated processes in early anaphase

Since cytokinesis was not responsible for the loss of PFGE chromosome bands in *top2-5*, we wondered whether transition through anaphase played any role. Anaphase starts when all sister kinetochores are attached to opposite spindle pole bodies through the microtubule-based spindle apparatus^31^. At that point, cohesion between sister chromatids is lost and the spindle pulls sisters apart. Once cells enter anaphase, the master cell cycle phosphatase Cdc14 is activated through the FEAR network to promote spindle elongation^32,33^, as well as resolution and condensation of the rDNA^34–37^. In addition, Cdc14 targets to the nucleoplasm and activates the structure-specific endonuclease (SSE) Yen1^38–40^, which can recognize and cut both Y-shaped and X-shaped molecules^38^. We reasoned that any of these Cdc14-mediated events could be responsible for the loss of chromosome bands observed ~ 120′ after the G1 release. However, the *top2-5 cdc14-1* strain was indistinguishable from *top2-5 cdc15-2*, ruling out this possibility (Figure S8).

### Loss of function of the structure-specific endonucleases Mms4-Mus81 and Yen1 worsens the *top2-5* PFGE shift

The late Rad52-independent X-shaped molecules we observed at the rDNA in *top2-5 cdc15-2* suggests that Top2 could prevent the formation of novel types of four-way DNA-DNA linkages. However, this takes place in the context of active SSEs that should have dealt with these branched structures. Assuming that there are more of these X-shaped linkages in chromosomes other than XII, a connection between these linkages and the late PFGE retention would require one out of these two possibilities: (i) late X-shaped molecules are refractory to SSEs (e.g., they could be hemicatenanes); or (ii) by-products of their processing by SSEs are responsible for the PFGE retention. In other words, the X-DNA that arises in *top2-5* could be cut by SSEs into another intermediate which, in turn, would be responsible for the late PFGE phenotype. Along with the Cdc14-regulated SSE Yen1, the complex Mus81-Mms4 cuts in metaphase the same anaphase bridge-prone X-DNAs than Yen1 cuts in anaphase^7^. Hence, we checked the PFGE shift in a strain deficient for both SSEs, and any other that may be regulated by Cdc14 in anaphase. However, the hypothesis that SSE by-products were responsible for the late PFGE shift was ruled out genetically through this *top2-5 cdc14-1 yen1Δ mms4Δ* mutant (Figure S9). On the contrary, this quadruple mutant showed more PFGE retention for all chromosomes at earlier time points (from 60′ onwards). Because we had not observed a PFGE retention in a *TOP2 yen1Δ mms4Δ* strain in a previous work^7^, it appears that the Top2 absence may indeed increase canonical X-shaped branched structures during S-phase, which are timely processed by SSEs, likely by Mus81-Mms4 as it is the one active before anaphase onset^41^. Nevertheless, the late PFGE shift is still present in active SSEs and it is not relieved in their absence. Altogether, we conclude that SSE activity is not responsible for the late PFGE shift.

### The* top2-5* structural chromosome change revealed by PFGE does not depend on the spindle force and cannot be reverted by re-activation of Top2

**Figure 5:**
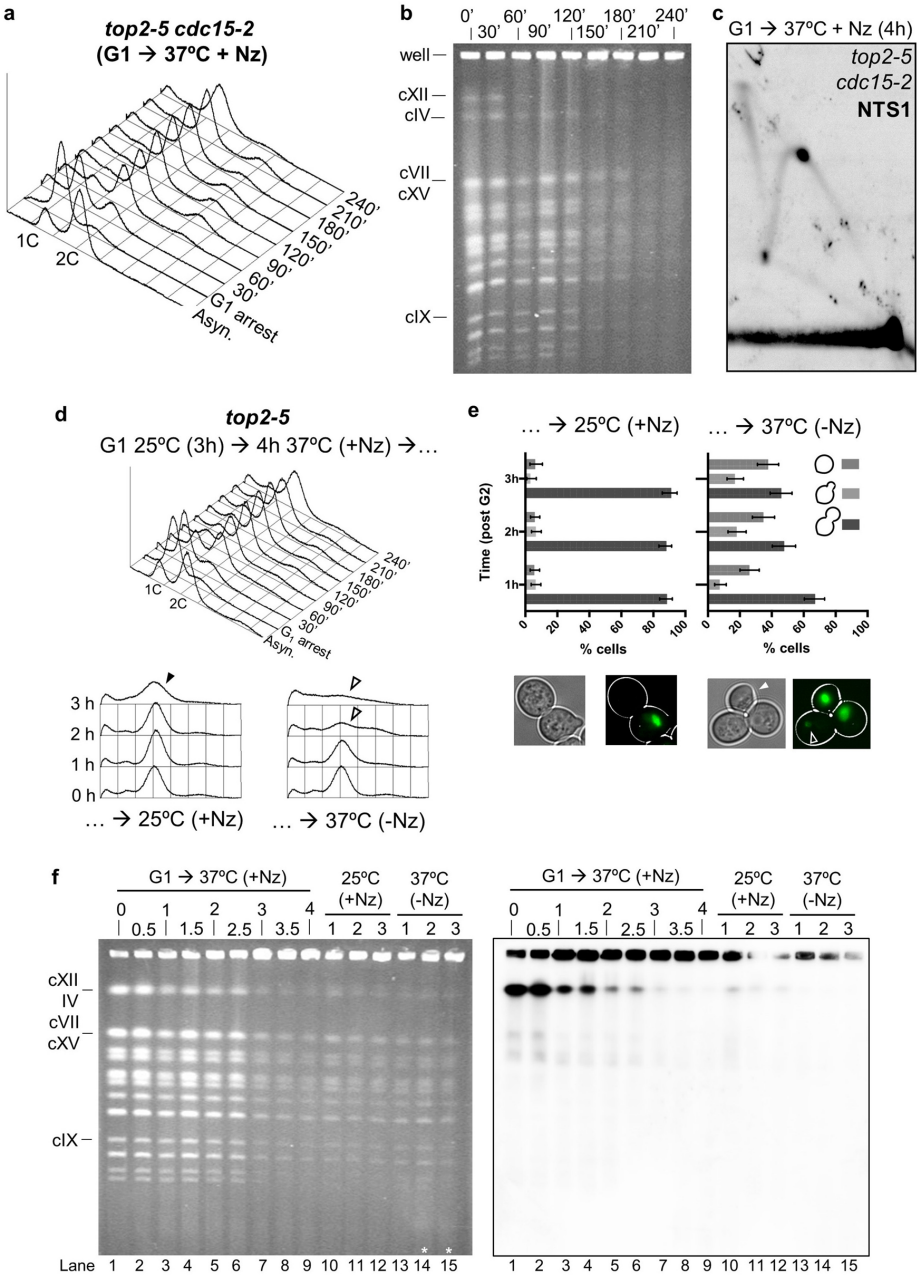
The late change in chromosome structure caused by the absence of Top2 is not a consequence of mitotic spindle forces, is not reversed by Top2, and does not trigger a G_2_/M checkpoint. (**a**–**c**) A synchronous G_1_ release experiment was performed for the *top2-5 cdc15-2* strain under restrictive conditions (37 ºC). At the time of the G1 release, Nocodazole (Nz) was added to depolymerize the spindle and block cells in G_2_/M. (**a**) FACS analysis of the DNA content during the first 4 h of the G_1_-to-Nz cell cycle. (**b**) EtBr-stained PFGE of the time course. (**c**) NN-2D for the rDNA NTS1 region 4 h after the G1 release. (**d**–**f**) A synchronous G_1_ release experiment was performed for the *top2-5* strain as in (**a**–**c**). After 4 h, the culture was split in two and incubated for another 3 h. One subculture was shifted back to 25 ºC (i.e., re-activate Top2) while keeping the Nz, whereas the other one was kept at 37 ºC while removing Nz. (**d**) FACS analysis of the DNA content during the first 4 h of the G_1_-to-Nz cell cycle (on the top) and during the extra 3 h after the culture splitting (underneath). (**e**) Cell morphology analysis during the extra 3 h incubations. Below the charts, representative micrographs of each subculture after 3 h; i.e., a mononucleated dumbbell versus a dumbbell with an uneven segregation (hollow arrowhead) plus a postcytokinetic G_1_-like cell (white arrowhead). (**f**) EtBr-stained PFGE of the whole experiment (left) and the corresponding Southern blot against the rDNA (right). Note how (i) the late PFGE general shift still occurs in Nz, (ii) how reactivation of Top2-5 does not reshape the chromosomes to enter the gel, and (iii) how the shift does not trigger a G_2_/M, so that a mitotic catastrophe still takes place after Nz removal (asterisks in lanes 14 and 15).

Neither cytokinesis nor Cdc14-controlled anaphase events were responsible for the PFGE phenotype, yet it clearly takes place in anaphase as determined by comparing microscopy and PFGE (Figs. 1, 2 and S8). We next checked whether the force of the mitotic spindle might trigger the loss of chromosome bands. Thus, we added nocodazole (Nz) to depolymerize the microtubules before *top2-5 cdc15-2* (Fig. 5a–c) and *top2-5 CDC15* (Fig. 5d–f) cells reached anaphase. Noteworthy, Nz also elicits the activation of the spindle assembly checkpoint^42^, leading to a transient cell cycle block at metaphase^43^. The FACS pattern we observed, with the corresponding long lasting 2C peak in both *top2-5* mutants (Fig. 5a,d, upper FACS profile), confirmed this arrest. The presence of > 95% of mononucleated dumbbell cells under the microscope (Fig. 5e, lower left) further confirmed the Nz arrest. Strikingly, even though spindle pulling forces were absent, the loss of chromosome bands still occurred (Fig. 5b,f, lanes 1–9). Likewise, the rDNA exhibited Y- and X-shaped intermediates (Fig. 5c).

Having found that the loss of chromosome bands can also happen in Nz-arrested cells after the absence of Top2, we next checked whether re-activation of Top2 could restore the chromosome bands. For that purpose, we shifted the temperature down to 25 ºC while maintaining the cells in Nz in the *top2-5* strain (Fig. 5d–f). Cells remained as mononucleated dumbbells with a 2C DNA content for at least 3 h after the temperature shift (Fig. 5d, lower left FACS profile; and Fig. 5e, left chart); however, this did not restore the structural integrity of the chromosomes (Fig. 5f, lanes 10–12). Since cultures were left in Nz for some time, and it is known that the G_2_/M arrest is leaky in Nz^43^, we repeated this experiment in a *top2-5 cdc15-2* with shorter incubation times (Figure S10). In this way, slippage from the Nz arrest was minimized while we further prevented entry into a second cell cycle by *cdc15-2*. We still observed that the PFGE shift was not modified after the temperature downshift. In addition, resuming both Top2 activity (25ºC downshift) and spindle forces (Nz removal) did not change the PFGE pattern either.

We conclude that the chromosome structural change upon Top2 depletion can occur in a G_2_/M block, is independent of spindle forces and is irreversible.

### G_2_/M checkpoints are blind to the *top2-5* structural chromosome change

The persistent loss of chromosome bands in Nz-arrested cells allowed us to assess whether the structural chromosome change triggers a G_2_/M checkpoint. This question could not be answered in cycling cells as the change happens in anaphase. However, once we observed the loss of chromosome bands in Nz, if a G_2_/M checkpoint were triggered we would see a delay in late cell cycle events after Nz removal in the *top2-5* strain. However, when Nz was removed we observed not only anaphase progression but also cytokinesis and the corresponding mitotic catastrophe (Fig. 5d, lower right FACS profile; and 5e, right chart).

In addition, we ruled out that the absence of a checkpoint response after the *top2-5* PFGE shift was a consequence of Top2 itself being a sensor/mediator of such checkpoint(s). Both hydroxyurea (HU) and methyl methanesulfonate (MMS) still arrested the *top2-5* strain in G_2_/M in restrictive conditions (Figure S11). HU creates replicative stress through formation of ssDNA behind the RFs, whereas MMS leads to RF stalling in addition to ssDNA gaps behind the RFs^5^.

### Dysfunctional mitochondria do not alter the PFGE shift in *top2-5*

Lastly, we checked whether the *top2-5* PFGE profile could be modulated by the pleiotropic effects observed in the absence of mitochondrial DNA (rho^0^ cells), which renders dysfunctional mitochondria. Mitochondria satisfy the high energy demands require for critical events during cell growth, including replication and condensation. We reasoned that a slower metabolism could slow down or prevent the PFGE shift. In addition, rho^0^ cells have altered dNTP pools and inefficient folding of enzymes require for DNA replication and repair^44^. Both deleterious effects may influence the PFGE outcome.

We found that a *top2-5* rho^0^ strain missegregates the histone-labelled nuclear mass to the same extend as its *top2-5* rho^+^ counterpart, also showing unequal segregation and a low percentage of visible anaphase bridges (Figure S12a). However, *top2-5* rho^0^ cells undergo a slower cell cycle, which includes a lengthened S-phase (Figure S12b, compare transposition of 1C to 2C content by FACS with that of Fig. 1b) and an apparent arrest in late anaphase (the 2C peak remains for at least 4 h). Remarkably, though, the postreplicative PFGE shift was still present (Figure S12c). The 30′ delay with respect to *top2-5* rho^+^ correlates well with the delay observed in the accumulation of the 2C content. We conclude those mitochondrial functions compromised in rho^0^ cells do not alter the PFGE shift.

### A GFP-based candidate screen of DNA damage and checkpoint proteins further indicates that *top2-5* cells do not detect the structural chromosome change as chromosomal damage

In addition to the previous set of experiments, we conducted a GFP-based screen of DNA damage proteins known to either form foci or increase their nuclear content upon DNA damage^45^. This screen was undertaken at the *top2-5 cdc15-2* block and included the Dpb11-yEmRFP as an additional reporter. Dpb11 is known to get enriched at certain types of anaphase bridges^46^. As a control, we included a *TOP2 cdc15-2* strain also blocked in telophase. We observed an increase in nuclear foci in the *top2-5 cdc15-2* block for the DNA replication stress markers Lcd1/Ddc2, Rfa2 and Rfa3, the latter two belonging to the ssDNA binding RPA complex (Table 1, Figures S13 and S14). The foci were predominantly present along the anaphase bridge. However, there were no differences for other important replication stress reporters such as Ddc1, Dpb11, Dna2 and Rad5. Importantly, there was no increase in foci of proteins involved in HR such as Rad51, Rad52 and Rad54. Altogether, we conclude that more ssDNA is present in cells blocked in telophase after passing through a cell cycle without Top2. However, this higher ssDNA level does not elicit an efficient DDR. Incidentally, there were RPA and Dna2 foci in *TOP2 cdc15-2* cells. Nearly 50% of telophase-blocked *TOP2 cdc15-2* cells had one Rfa2/3 focus.

**Table 1:** Location pattern in telophase-blocked cells of DNA damage checkpoint and repair proteins after going through a synchronous cell cycle with or without Top2.

Protein^a^	Lab#1^b^	Lab#2^c^			At the telophase block (37ºC × 3 h)	
	As	As	HU	MMS	*top2-5 cdc15-2*	*TOP2 cdc15-2*
Rfa2	+	+	+	++	+++ (S)	++
Rfa3	+	+	+	++	+++ (S)	++
Lcd1 (Ddc2)	−	+	++ (f)	++	++ (29%); NAI	−
Ddc1	−	−	++ (f)	++	−	−
Dbp11	−	−	−	++	−	−
Dna2	−	+	−	+	+	+
Elg1	−	−	− ; NAI	− ; NAI	−	−
Mrc1	−	+ (f)	+ (f)	−	−	−
Ctf18	−	−	+ (f)	−	−	n.d. (w)^d^
Cdc28	−	+	+	+	−	−
Rad9	−	−	−	+ (f)	−	−
Rad53	−	−	+ (f)	++ (f)	−	−
Chk1	−	−	− ; NLI	− ; NLI	−	−
Dbf4	−	−	− ; NAI	− ; NAI	−	−
Cdc7	−	−	− ; NAI	− ; NAI	−	−
Dun1	−	−	−	− ; NAI	−	−
Rad5	−	−	+ (f)	++ (f)	−	−
Rad6	−	−	−	−	−	−
Rad50	−	−	−	−	−	−
Xrs2	−	−	+ (f)	+ (f)	−	n.d. (w)
Yku70	−	−	−	−	−	n.d. (w)
Yku80	−	−	−	−	−	−
Irc20	−	−	−	−	−	−
Exo1	−	−	++ (f)	−	−	−
Sae2	−	++ (f)	++ (f)	++ (f)	−	−
Rad51	−	−	−	−	−	−
Rad52	−	−	−	−	+ (6.4%)	+ (6.5%)
Rad54	−	−	−	++	−	−
Rad55	−	−	−	−	−	−
Rad57	−	−	−	−	−	−
Rad59	−	−	−	+	−	−
Rad10	−	−	+ (f)	− ; NAI	−	−
Cdc13	−	−	−	+	−	−
Mms21	−	+	+	+	−	−
Nse4	−	+	+	+	−	−
Esc2	−	−	− ; NAI	− ; NAI	−	−
Mph1	−	−	−	+ (f)	−	−
Rmi1	−	−	−	−	−	−
Slx4	+ (f)	+	++ (f)	++ (f)	−	−

^a^Proteins are sorted according to the approximate order of action after replication stress^5^. Most proteins inform about replication stress (or DNA damage) by the formation of nuclear foci. Other proteins may concentrate in the nucleus upon replication stress (e.g., Elg1; Chk1; Dbf4-Cdc7; Dun1; Esc2). Cells with foci? −, < 5%; + , 5–25%; ++, 25–60%; +++ , > 60%. In some instances, the actual percentage is between brackets. Foci intensity? (f), faint; (S), strong. Location/abundance? NLI, higher cell number with nuclear location; NAI, nuclear abundance increased.

^b^Lab#1: As seen at https://yeastgfp.yeastgenome.org/.

^c^Lab#2: As seen at http://images.yeastrc.org/tkach_brown/replication_stress.

^d^n.d. (w) = not determined because wrong protein location.

## Discussion

Top2 is the critical enzyme that removes sister chromatid intertwines prior to chromosome segregation in anaphase. The absence of Top2 is broadly recognized to lead to a type of mitotic catastrophe whereby anaphase bridges, which arise from these intertwined chromatids, are severed during cytokinesis. Altogether, the results shown in this paper confirmed that *top2-5* also leads to a mitotic catastrophe; we here demonstrate this by microscopy, complementing our previous work^15,23^, but also by FACS and by PFGE. The mitotic catastrophe in *top2-5* seems greater than other previously studied *top2-ts* alleles, at least by FACS analysis and microscopy^15,17,47^, and might be a feature of either the *top2-5* allele itself or the genetic background it is in^15^. However, the most shocking *top2-5* phenotype we introduce here is the disappearance of chromosome bands in a PFGE at a stage prior to the mitotic catastrophe. The overall DNA content we quantified by FACS, as well as the absence of in-gel smears and broken rDNA in the Southern blots, strongly suggests that the disappearance of bands correlates with entrapment of chromosomes in the loading well (Figs. 1, 2, 5, S4–S6). Moreover, DNA release from the plug was accomplished by digestion with BglII for the NN-2D analysis (Figs. 3 and 4). We distinguished two classes of chromosomes affected by the PFGE retention: the rDNA-bearing chromosome XII and all the other 15 chromosomes. The former persistently gets well-bound from the beginning of the S phase, which is compatible with, at least, unfinished replication (protracted Y-shaped DNA at the RFB in *top2-5 cdc15-2*; Fig. 3, 4 and 5). By contrast, chromosomes other than XII manifest the same PFGE behaviour much later in the cell cycle; after replication appears to have ended by both FACS and PFGE (Figs. 1, 2, 4, 5, S1, S3–S6, S8 and S12). A closer look at this late PFGE behaviour suggests that it might be independent of transition between cell cycle stages (i.e., S-phase to metaphase to anaphase) and the key spatial and molecular changes that occur in such transitions (e.g., activation of structure-specific endonucleases, spindle pulling forces, hypercondensation by Cdc14, etc.)^2,48^. Rather, it seems there is a fixed time window between the end of S-phase (as determined by FACS) and the PFGE shift (~ 1–2 h). Thus, in synchronous cell cycle cultures the loss of chromosome bands takes place well within anaphase (Figs. 1, 2 and S8), and the loss of chromosome bands is present in Nz-treated cells as well (Fig. 5 and S10). Importantly, these latter experiments led us to conclude two important features of the loss of chromosome bands in PFGE; (i) no G_2_/M checkpoint is activated after the loss of chromosome bands; and (ii) the loss of chromosome bands is irreversible with respect to Top2 activity.

There are four known causes of chromosomes entrapment in the loading well during PFGE: linear chromosomes larger than 10 Mbp^49^, relaxed circular chromosomes larger than 100 kbp^50^, the presence of chromosomes with branched structures^7,24,25,51^, and the presence of large portions of ssDNA^52,53^. Branched structures and ssDNA physiologically arise during replication and DNA repair through HR. Whereas a theoretical and experimental framework exists to explain the relationship between PFGE trapping and chromosome size^54^, the causes of why chromosomes carrying DNA branches or ssDNA gaps do not enter PFGE remain undetermined. Because of this lack of knowledge on the PFGE technique, we cannot fully draw at present the postreplicative pathway to whatever structures preclude the affected chromosomes from entering the PFGE. From results we present in this work (Figs. 3, S13 and S14; Table 1), it appears that both ssDNA and replicative branched structures are already present in chromosomes that, nonetheless, enter a PFGE. For instance, gaps of unreplicated DNA were observed by combing in the *TOP2 cdc15-2* block (Fig. 3). Although this may reflect a limitation of the combing technique for the purpose of quantifying underreplication, it is remarkable that a recent report shows that chromosomes are not fully replicated in a *TOP2 cdc15-2* block^55^. Likewise, we have shown that sister chromatids are somehow connected with each other in the *TOP2 cdc15-2* block and form retrograde anaphase bridges after DSBs^56^. In addition, cytological markers of ssDNA and replication stress are present in the *TOP2 cdc15-2* block, although there is an increase in the *top2-5 cdc15-2* block (Table 1; Figures S13 and S14). One of these markers, the RPA complex, has been recently seen by others in the *TOP2 cdc15-2* block^55^. Because we could not see a difference in the amount of underreplicated material between *TOP2 cdc15-2* and *top2-5 cdc15-2* (Fig. 3), we speculate that a late modification of remaining Y-shaped branches in the absence of Top2 might trigger the PFGE shift. This modification would not be sensed as DNA damage (Figs. 1, 2, 5 and S11) and would not depend on Rad52-driven HR (Fig. 4). An interesting possibility is the eventual regression of converging RFs into four-way HJ-like chicken foot structures (Figure S15a), which are particularly enriched when cells cannot sense RF problems^57^. This scenario is compatible with the presence of Rad52-independent NN-2D X-shaped signals we observed in the rDNA (Fig. 4). Moreover, RF regression is expected for *top2* mutants as positive supercoiling accumulates ahead of the converging RFs at replication termini^58,59^. Where are these persistent RFs? We have shown that the RFB at the rDNA locus is one of these (Figs. 3, 4 and 5), but this only accounts for chromosome XII. We envisage that other difficult-to-replicate regions shared by all chromosomes may be involved. These regions would include centromeres, G-quadruplex, fragile sites, transposable elements, non-coding RNAs and subtelomeric regions^18,55,60–62^. However, we checked one of such regions, the replication temini locus *TER302* in chromosome III, but could not detect persistent RFs in *top2-5 cdc15-*2. Recently, another paper has shown that pericentromeric regions accumulate DNA damage markers during S-phase^63^. Again, this damage does not appear to arrest the cell cycle^64^. A corollary of these assertions is that the traditional claim for ongoing replication as one cause of chromosome entrapment during PFGE should be revised. Perhaps, chromosomes get entrapped not by having RFs but by ensuing RF modifications. Alternatively, cumulative topological stress could promote dsDNA unwinding towards ssDNA that, followed by ectopic or sister re-annealing, would create four-way DNA origamis. In addition, ssDNA tracks may be interlocked by type IA topoisomerases (Top3), forming hemicatenanes. These solutions are indeed compatible with the presence of more ssDNA in *top2-5 cdc15-2* while not observing larger underreplication gaps than the control *TOP2 cdc15-2*. In addition, it predicts Rad52-independent X-shaped molecules at the rDNA and lack of sensing by the DNA damage checkpoints. Another possibility compatible with more ssDNA tracks but the same levels of underreplication is partial re-replication taking place in the absence of Top2. Perhaps, the perturbed topology at the replication origin deregulates how many times they can fire within a single cell cycle. However, it is difficult to envisage how re-replication can proceed in such topological jumble without triggering a DNA damage checkpoint.

Finally, we cannot rule other explanations for the PFGE shift such as massive topological intertwinings, or even knots, between different chromosomes after prolonged Top2 absence (Figure S15b). Catenations between the replicated sister chromatids is the immediate consequence of the Top2 absence; however, within the constrained space of the nucleus, and with other important dynamic events taking place in G_2_/M/anaphase, such as transcription and condensation, it is difficult to envisage how cells avoid cumulative topological problems without Top2. Of note, previous studies with *top2-ts* showed vastly interlocked/knotted plasmids^16,17,65^. Massive interchromosomal intertwinings/knotting may form a chromosome web that trap chromosomes. The presence of persistent late replication intermediates and complex topological intertwinings as explanations of the PFGE are not mutually exclusive. Indeed, knotting and hemicatenanes have been related to failure to finish replication^66,67^.

In conclusion, the lack of Top2 postreplicatively modifies the structure of all yeast chromosomes in a way that diminish their ability to run in a PFGE. Future work on the actual physical nature of the DNA molecules trapped in the PFGE, together with genetic screenings of modifiers, should clarify this late chromosome structural change and perhaps assign a novel role for Top2 in the chromosome biology field.

## Materials and methods

### Yeast strains and experimental conditions

All the strains used in this work are listed in Table S1. All strains were grown overnight in air orbital incubators at 25 °C in YEPD. Time course experiments were performed as follows: asynchronous cultures were adjusted to OD_600_ = 0.3 and then synchronized in G1 at 25 °C for 3 h by adding 50 ng/ml of alpha-factor. The G_1_ release was induced by washing the cells twice and resuspending them in fresh media containing 0.1 mg/ml of pronase E. Then, they were incubated at 37 °C for 3–4 h. In time course experiments, samples were taken every 30 min for microscopy, FACS and PFGE.

### Microscopy and flow cytometry (FACS)

H2A2-GFP was analyzed by wide-field fluorescence microscopy as reported before^15^. Briefly, series of z-focal plane images (15–20 planes, 0.15–0.3 μm depth) were collected on a Leica DMI6000, using a 63×/1.30 immersion objective and an ultrasensitive DFC 350 digital camera, and processed with the AF6000 software (Leica). Scale bars in micrographs depict 5 μm; BF stands for bright field.

DNA content by flow cytometry analysis (FACS) was done as previously described using a BD FACScalibur equipment^7^. An asynchronous culture of each strain growing at permissive temperature was used to calibrate the 1C and 2C peaks before reading the samples. Strains carrying the *cdc15-2* and *cdc14-1* alleles rendered a minor 2C peak at the G1 arrest. This peak corresponded to 10–20% of G1 samples and comprised cells responsive to alpha-factor but that remain attached as pairs (Figure S7). This phenotype likely stems from delayed septation at permissive temperatures. This minor 2C transposes into a 4C peak when cells are released from the G1 arrest, giving the false impression of re-replication.

### Pulsed-field gel electrophoresis (PFGE) and neutral, neutral two-dimensional electrophoresis (NN-2D)

Yeast DNA for PFGE and NN-2D was extracted in low-melting agarose (LMA) plugs in conditions known to avoid branch migration^7^. Briefly, 4 OD_600_ equivalents were embedded into a 0.5% (w/v) LMA plug and then digested with zymolyase (2 units, day 1), RNaseA (10 µg/ml; day 1) and Proteinase K (1 mg/ml; day 2). All digestions were carried out at 37 °C (including the Proteinase K step). Yeast DNA for combing analysis was also extracted in LMA but in different conditions (see below). In case of subsequent overnight treatment of the agarose plugs with 250 units of BglII (R0144M, New England Biolabs), Proteinase K was first inactivated with 1 mg/ml Pefabloc (11429868001, Roche).

PFGEs were carried out using a CHEF DR-III system (Bio-Rad) and we followed standard conditions with minor modifications^7^: 1% agarose gel in 0.5× TBE buffer and run at 14 °C for 40 h at 5 V/cm with an initial switching time of 47 s, a final of 170 s, and an angle of 120°. The chromosome bands in the gels were visualized with EtBr staining. For Southern blots we used a fluorescein-labelled probe (Sigma-Aldrich, #11585622910) against the NTS1 region within the rDNA^7,26^. Detection was performed by chemiluminescence (Amersham Hyperfilm ECL film) using an anti-fluorescein antibody coupled to alkaline phosphatase (Sigma-Aldrich, #11426338910) and using CDP-star (Sigma-Aldrich, #11759051001) as the substrate.

NN-2Ds for NTS1 were also performed from agarose plugs. These were digested with BglII, run in two dimensions, and transferred to positively charged nylon membranes for Southern analysis as described before^7,26^. Comparison between samples was based on expositions that render equivalent 1N/2N linear spots. The NN-2Ds for *TER302* was carried out according to the CTAB DNA extraction protocol^18^, as 1N and 2N spots from this single-copy genomic region were not strong enough from plugs.

### DNA combing and immunodetection

DNA combing experiments were performed as described before^68^. Firstly, *top2-5* and *cdc15-2* alleles were transferred by PCR methods to a strain suitable for BrdU DNA labelling (Table S1)^28^. The resulting strains were first arrested in G1 at 25 °C by incubating with 5 µg/mL alpha-factor (*BAR1* genotype) for 3 h. Cells were then released from G_1_ by adding Pronase E (100 µg/mL final concentration) and incubated 4 h at 37 °C. Half an hour before Pronase addition, BrdU was added to the cultures at 400 µg/mL final concentration. Samples were taken 4 h after the release and immediately treated with 0.1% w/v sodium azide and cooled down to 0 °C. DNA for combing was extracted in LMA plugs. Relative to the plugs prepared for PFGE and NN-2Ds, these plugs used the following settings: 4 × 10^7^ cells (~ 1 OD_600_ equivalents), 0.5 U Zymolyase (2 days of incubation), no RNAse, and 3 days incubation with Proteinase K at 50 °C.

For DNA combing, the plugs were incubated at RT for 30 min with 1 μL of YOYO-1 (Molecular Probes, Y3601) in 150 μL of TE50 buffer (10 mMTris-HCl pH 7.0, 50 mM EDTA). Then, they were washed 3 times in TE50 buffer, and incubated twice for 5 min in 2 mL MES buffer (7:3 v/v MES hydrate:MES sodium salt 50 mM pH 5.7). After that, the plugs were melted at 72 °C for 20 min. The solution was transferred to 42 °C for 15 min and incubated overnight in 3 U of β-agarase I (New England Biolabs, M0392). Next, the solution was heated to 72 °C for 10 min, cooled to room temperature (RT) and poured into the reservoir of the combing device. The silanized combing coverslips were incubated into the solution for at least 5 min, and then pulled out at a constant speed of 710 μm/s. Finally, they were incubated for 90 min at 60 °C, and mounted on a glass slide.

For immunodetection, the slides were dehydrated by sequential 5 min incubations with 70%, 90%, and 100% v/v ethanol at RT. They were air dried and the DNA was denatured in 1 M NaOH for 25 min at RT. Next, the slides were washed 5 times in PBS and incubated for 5 min in PBS-T (PBS plus 0.05% v/v Tween-20). After that, 21 μL of blocking buffer (PBS-T plus 10% w/v BSA) were added and dispersed on the coverslip and the slides were incubated in a humidity chamber for 30 min at 37 °C. Next, the coverslips were removed by dipping the slides into PBS-T, and 21 μL of anti-BrdU solution (1:40 dilution in blocking buffer of the rat anti-BrdU antibody [AbD Serotec, MCA2060]) were added, dispersed and incubated (1 h) as the previous step. The same steps were carried out after that with the anti-DNA solution (1:50 dilution of the anti-DNA antibody [Millipore, MAB3034]) and the secondary antibodies solution (1:75 dilutions of Alexa Fluor 488 goat anti-rat and Alexa Fluor 546 goat anti-mouse [Molecular probes, A11006 & A11030, respectively]), always washing with PBS-T in between. Finally, 10 μL of ProLong Gold antifade reagent (Molecular Probes, 36930) were added and the samples were covered with a fresh coverslip.

Only DNA fibers larger than 100 kbp and markedly labelled with BrdU were included in the analyses. A low number of fibers (1% in *TOP2 cdc15-2* and 9% in *top2-5 cdc15-2*) were wholly devoid of BrdU. We considered that these fibers came from cells that did not enter S-phase after the G_1_ release, as we previously showed that ~ 10% of the *top2-5* progeny at 25 °C may be inviable^23^.

### Mini-screening of fluorescent DNA stress markers

The GFP-fusion library (Invitrogen, catalog number: 95702) was used as the basis for the mini-screening^69^. To introduce both the *top2-5* and *cdc15-2* alleles into the library, a series of PCR-based transformations were carried out to obtain the strain OQR84. This strain was also designed to carry the anaphase bridge reporter Dpb11-yEmRFP. The strain OQR84 was crossed with preselected strains from the GFP-fusion library and then haploids were selected for two genotypes: *DPB11-yEmRFP gene-GFP TOP2 cdc15-2* and *DPB11-yEmRFP gene-GFP top2-5 cdc15-2* (Table S1).

To carry out the screen, cells were grown overnight in 96-well plates at permissive temperature (25 °C) in synthetic complete medium supplemented with 100 µg/ml adenine. The following day, cells were diluted 1/10 and grown for 2 h at 25 °C. Next, cells were incubated at restrictive temperature (37 °C) for 3 h and finally transferred to 384-well CellCarrier plates (PerkinElmer, 6007550) for imaging on an Opera QEHS high-content screening microscope (PerkinElmer). Single planes of 5 different fields were taken per each strain of the library using a 60× water immersion objective and CCD cameras with the proper filter sets to visualise either GFP or RFP. The exposure time for each channel was 1 s. Data analysis to determine the presence of GFP-tagged proteins on Dpb11-labeled anaphase bridges was performed manually. Foci number was also determined by eye.

## Supplementary Information

Supplementary Information.

## Data Availability

The datasets supporting the conclusions of this article are included within the article and its additional files.
