# Supplementary material for: Topoisomerase II deficiency leads to a postreplicative structural shift in all Saccharomyces cerevisiae chromosomes
Source: Sci Rep. 2021 Jul 22;11:14940. doi: 10.1038/s41598-021-93875-5 (PMC8298500; doi:10.1038/s41598-021-93875-5)
Supplement: Supplementary file 1 — Supplementary Information. [file 41598_2021_93875_MOESM1_ESM.pdf]

**Topoisomerase II deficiency leads to a postreplicative structural shift in all *Saccharomyces cerevisiae* chromosomes.**

Jessel Ayra-Plasencia[1,2,a], Cristina Ramos-Pérez[1,3,b], Silvia Santana-Sosa[1,2], Oliver Quevedo[4,c], Sara Medina-Suarez[1,2], Emiliano Matos-Perdomo[1,2], Marcos Zamora-Dorta[1], Grant W Brown[3], Michael Lisby[4] and Félix Machín[1,5,6*]

[1] Unidad de Investigación, Hospital Universitario Nuestra Señora de Candelaria, Tenerife, Spain.

[2] Escuela de Doctorado y Estudios de Postgrado, Universidad de La Laguna, Tenerife, Spain

[3] Department of Biochemistry and Donnelly Centre, University of Toronto, Canada

[4] Department of Biology, University of Copenhagen, Denmark.

[5] Instituto de Tecnologías Biomédicas, Universidad de la Laguna, Tenerife, Spain.

[6] Facultad de Ciencias de la Salud, Universidad Fernando Pessoa Canarias, Las Palmas de Gran Canaria, Spain.

[a] Present address: CEAMED S.A., Tenerife, Spain.

[b] Present address: BenchSci Analytics Inc., Toronto, Canada.

[c] Present address: River Stone Biotech, Copenhagen, Denmark.

* Corresponding author: Félix Machín. Unidad de Investigación, Hospital Universitario Nuestra Señora de la Candelaria, Ctra del Rosario 145, 38010 Santa Cruz de Tenerife, Spain. Tfno: +34 922 602 951. E-mail: fmachin@fciisc.es

SUPPLEMENTAL MATERIAL

**SUPPLEMENTAL FIGURES.**

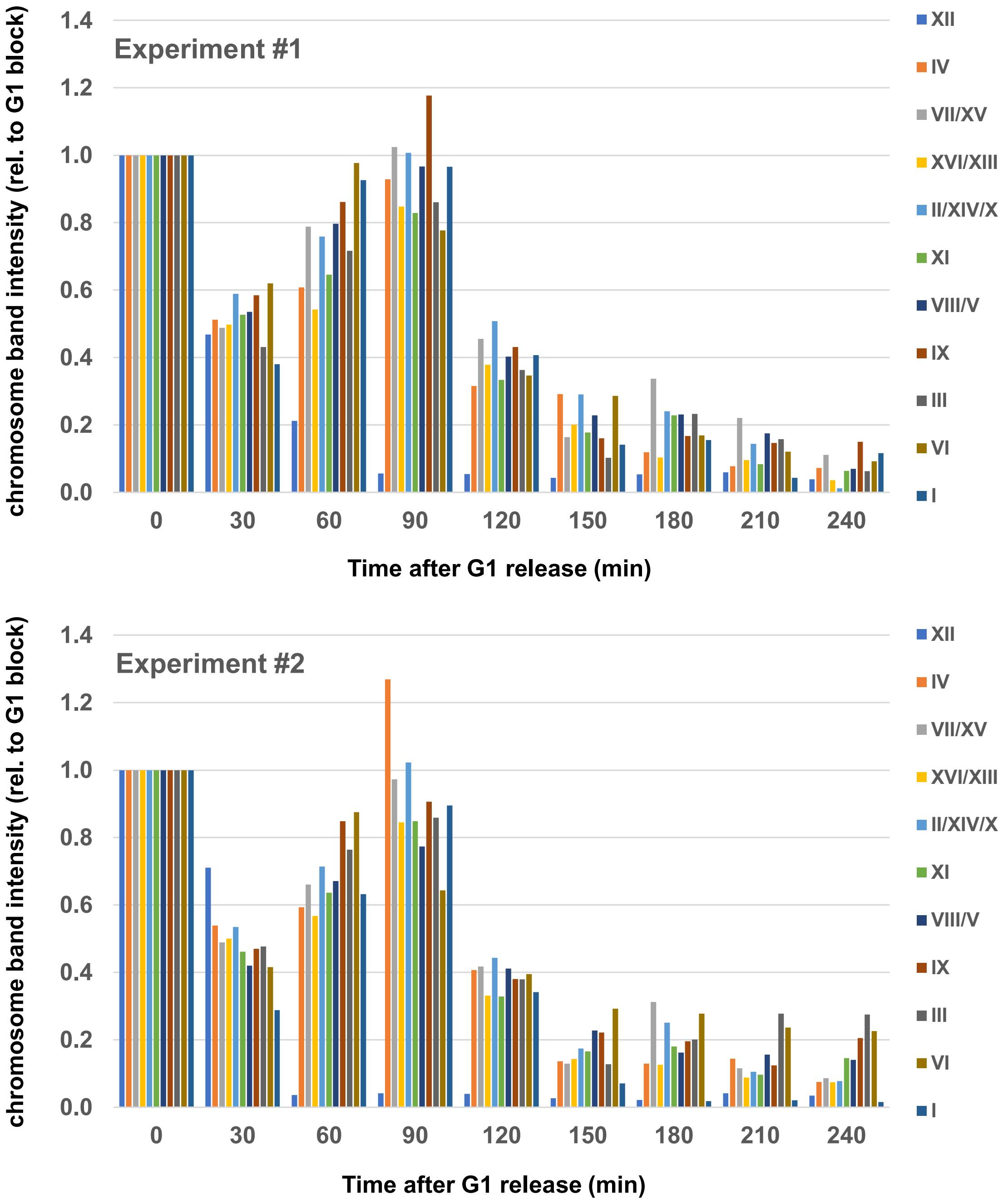


**Figure S1. Quantification of PFG chromosome bands after the *top2-5* release from G1.** After the PFGEs obtained from time course experiments as shown in Figure 1, gels were stained with ethidium bromide, photographed, each band quantified by Fiji/ImageJ (https://imagej.net/Fiji), and represented against the band value at G1. The experiment #1 corresponds to the PFGE shown in Figure 1e.

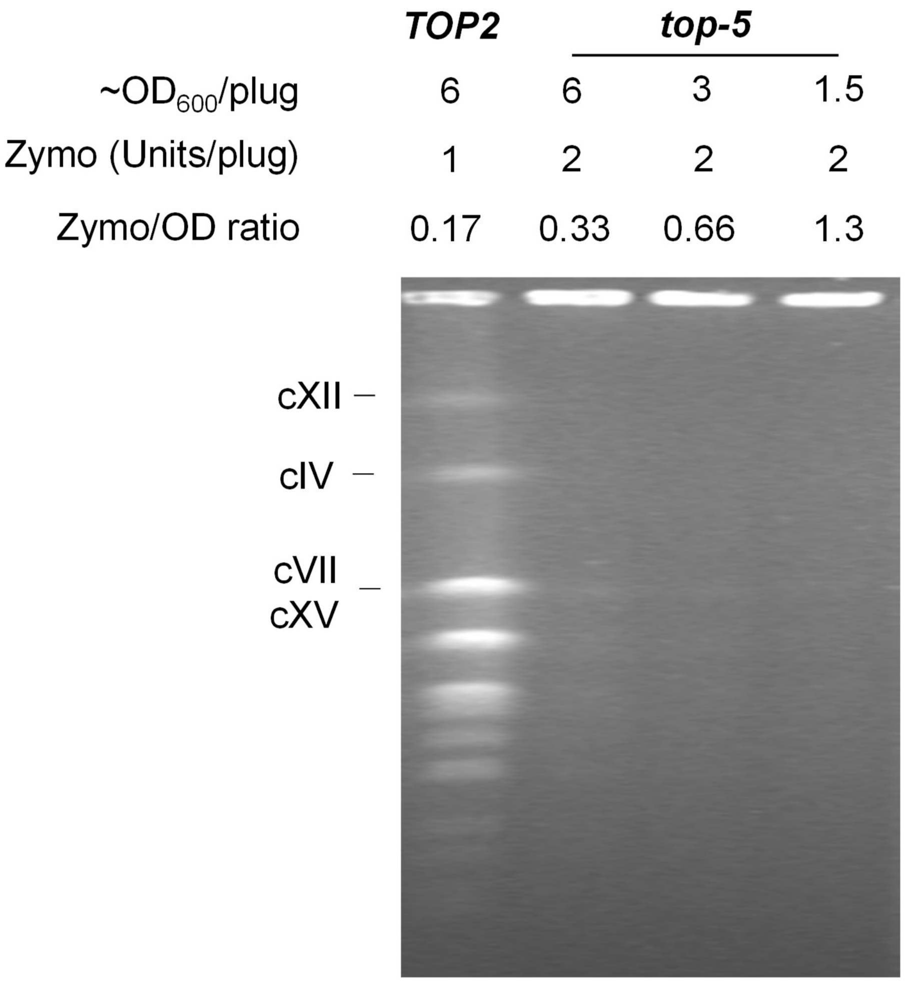


**Figure S2. Well entrapment of *top2-5* is not due to special wall digestion requirements in this mutant.** A time course experiment was carried out as stated in Figure 1. Four hours after the G1 release, samples from the *TOP2* and *top2-5* strains were taken from plug preparation under different cell wall digestion treatments. In general, the number of cells ($OD_{600}$ equivalents) and the amount of zymolyase were changed. The standard values in all other plug preparations of this article were 4 $OD_{600}$ and 2 units zymolyase. Note how increasing ODs and decreasing zymolyase unit in *TOP2* plugs still allowed chromosome migration, whereas decreasing ODs in *top2-5* did not ameliorate chromosome entrapment. The image of the PFG is vertically squeezed to half of its actual length.

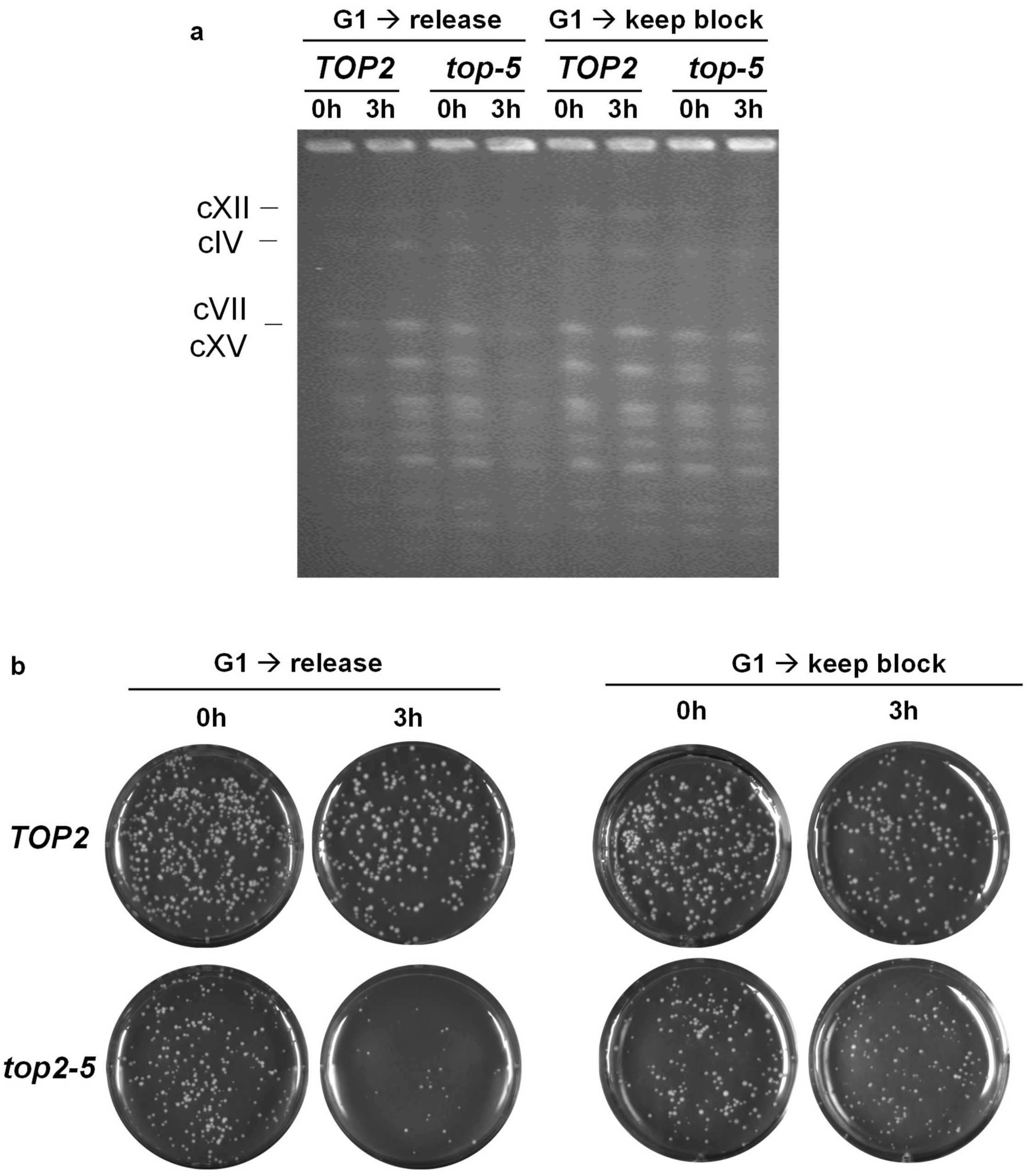


**Figure S3. Maintaining the G1 block prevents chromosomes from well-entrapment in *top2-5*.** The *TOP2* and *top2-5* strains were arrested in G1 for 3h at 25 ºC. Then the cultures were split in two, one of them was released into a synchronous cycle (G1 → release) and the second one was kept in the G1 block. All four cultures were incubated at 37 ºC for 3 more hours before stopping the experiment. At the time of the G1 block at 25 ºC and after 3h at 37 ºC, samples were taken for PFGE and assessment of clonogenic survivability. (**a**) PFGE. The image of the PFG is vertically squeezed to half of its actual length. Note that chromosomes do not get entrapped in *top2-5* cells kept in G1. (**b**) Clonogenic survivability. Note that *top2-5* cells do not loose viability when kept in G1.

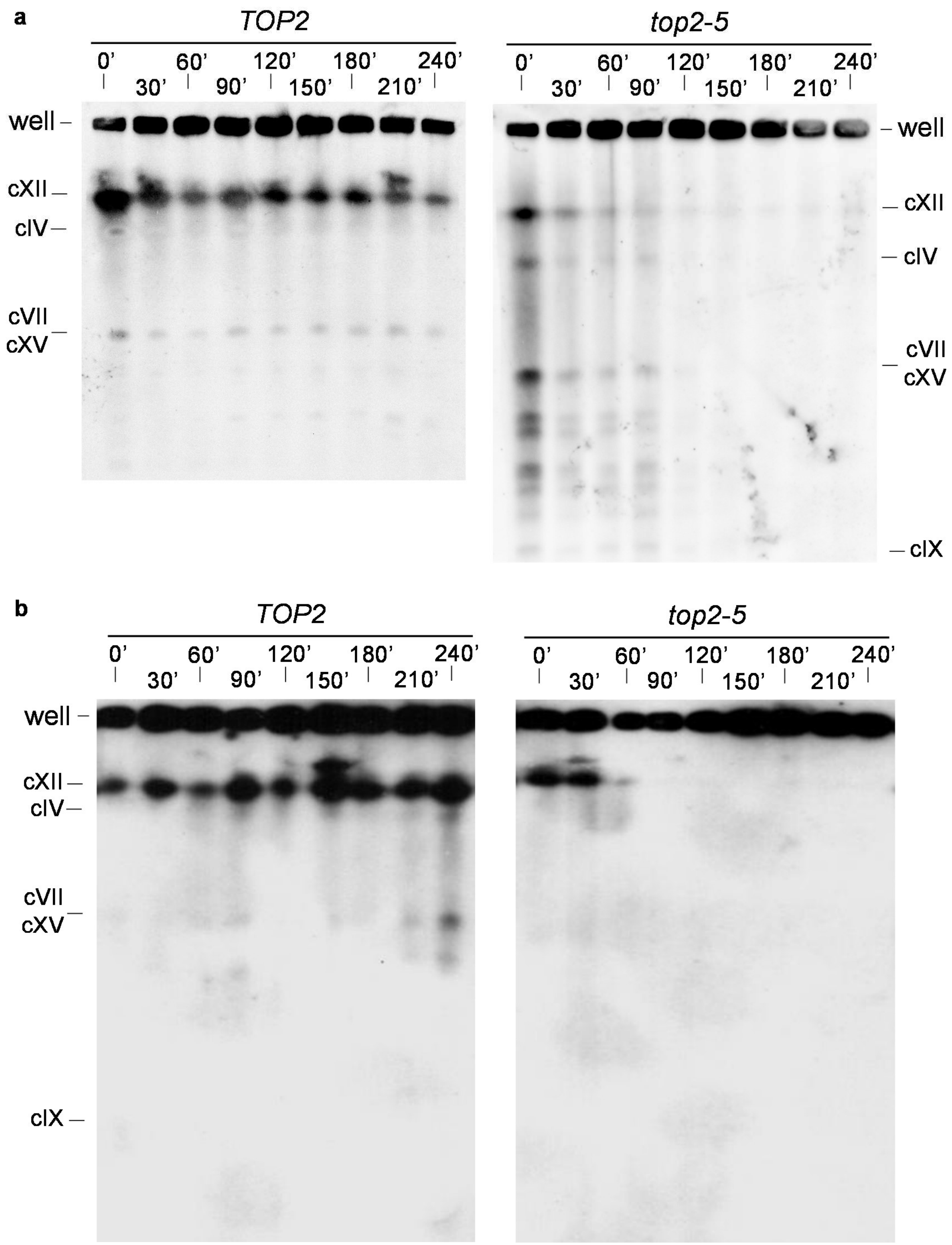


**Figure S4. No sign of fast-migrating broken chromosomes after Top2-5 inactivation.** Two synchronous G1 release experiments were performed at 37 ºC for isogenic *TOP2* and *top2-5* strains. At the indicated time points, samples were taken for microscopy, FACS and PFGE (shown in Figure 1 for experiment #1). After PFGEs, Southern blots were undertaken with probes against the NTS1 region of the rDNA (chromosome XII, cXII). (a) Southern blots from experiment #1 (*top2-5* under lower stringency conditions). (b) Southern blots from experiment #2. Each experiment used independent NTS1 probes.

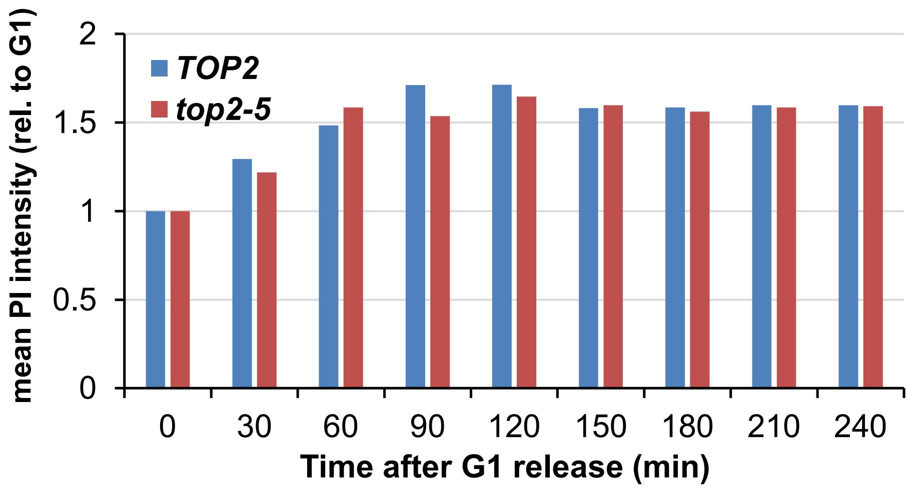


**Figure S5. Quantification of the average DNA content after the *top2-5* G1 release.** Area under the curve was quantified for the FACS profiles shown in Figure 1d. Note that *TOP2* and *top2-5* have an equivalent DNA content during the time course despite the extremely flattened profile of *top2-5* at the latest time points (180'-240'). The values never reached the theoretical 2xG1 content, probably because propidium iodine stains budded cells less efficiently.

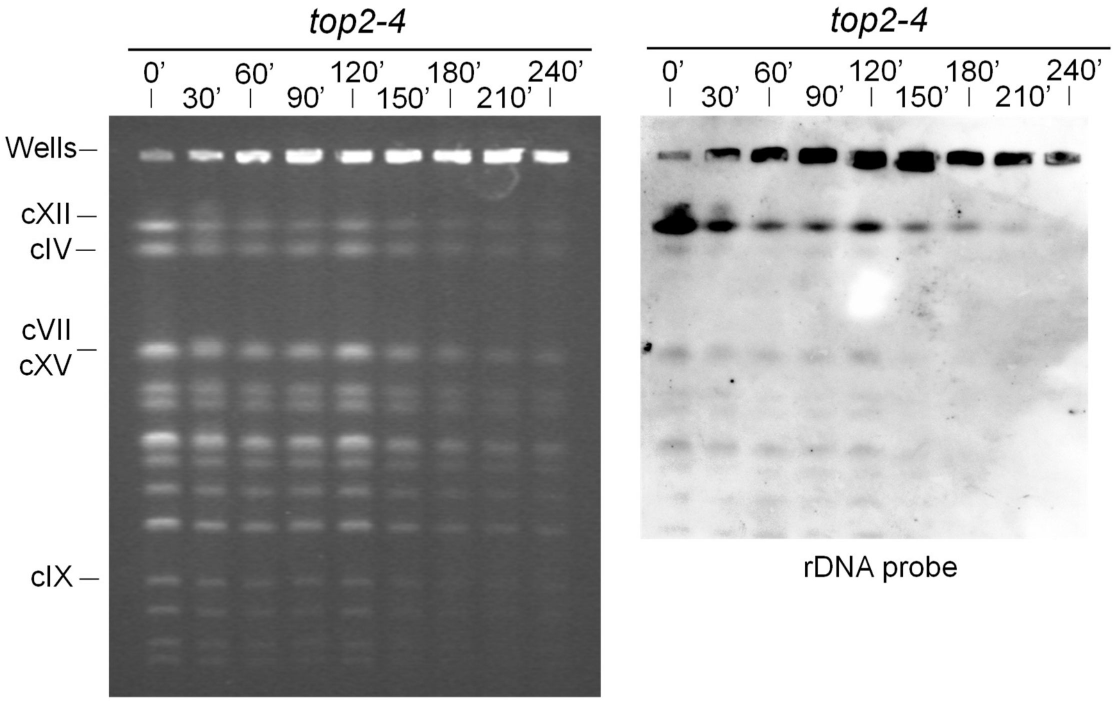


**Figure S6. The mutant *top2-4* also entraps chromosomes after the mitotic catastrophe.** An isogenic strain carrying the *top2-4* ts allele was subjected to the same experiment shown in Figure 1. A PFGE was run and a Southern blot against the rDNA was performed in the same conditions. Note how *top2-4* led to entrapment from 150' onwards, although the phenotype was slightly milder, including for chromosome XII.

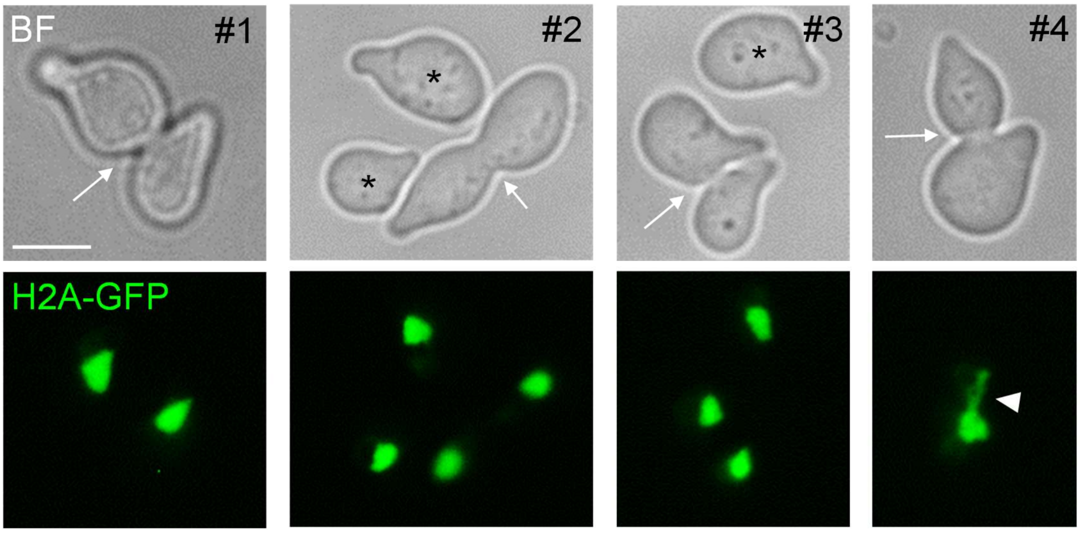


**Figure S7. Incomplete cytokinesis and septation in cdc15-2 derivatives of *top2-5* and *TOP2* blocked in G1.** Four representative micrographs are shown from the Figure 2 experiment. Note how G1-blocked cells (they all responded to alpha-factor as reported by the characteristic pear-like morphology) can appear as either singlets (asterisks) or doublets. The latter stems from incomplete septation (white arrows) and/or cytokinesis in the previous cell cycle. At least in the example #4, a defect in cytokinesis is evident since the doublet shows a chromatin bridge (white arrowhead in the GFP channel). These doublets likely account for the minor 2C peaks observed at time 0 in the corresponding FACS, and the ensuing minor 4C peaks during the release. The scale bar depicts 5 µm. BF, bright field.

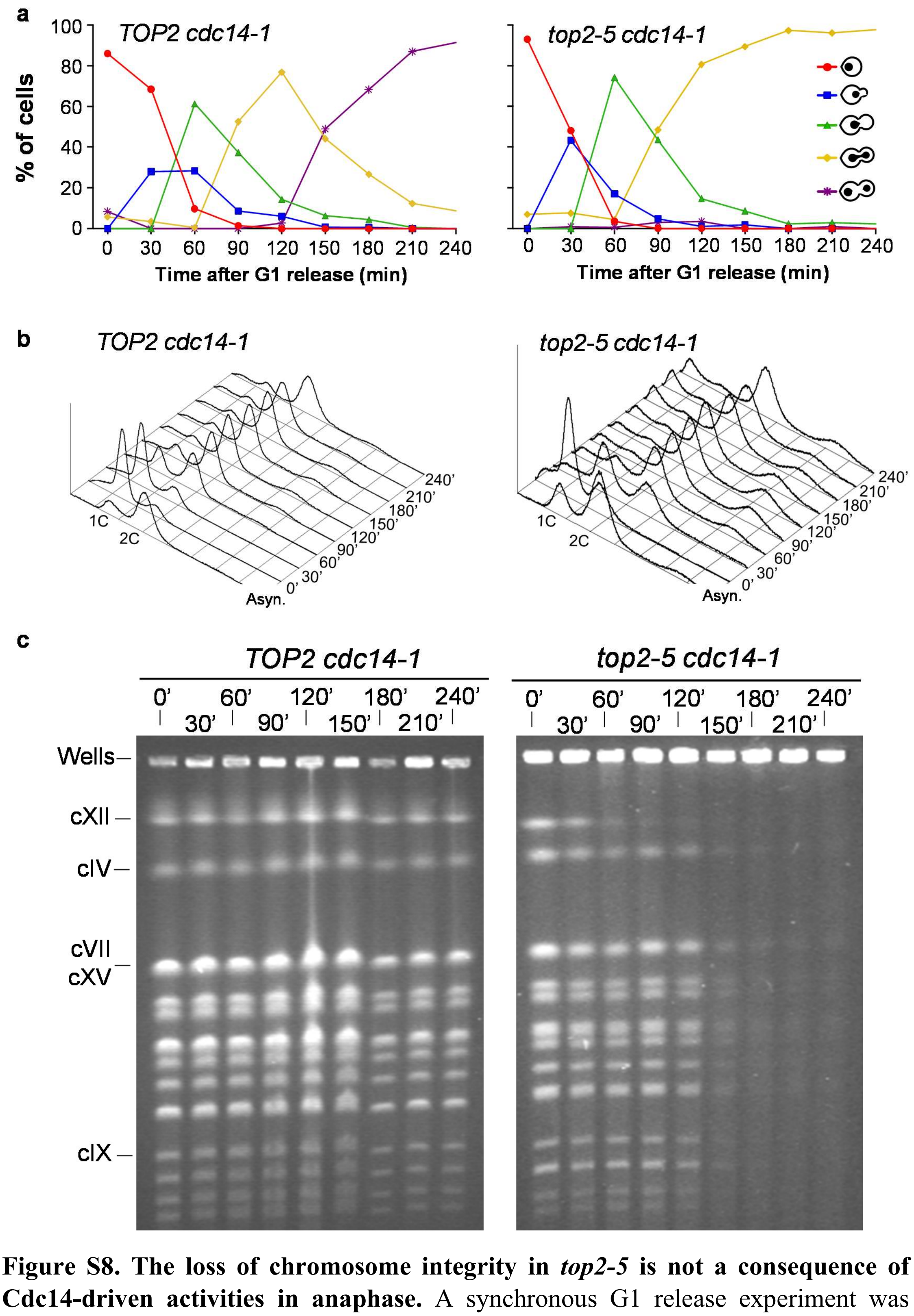


**Figure S8. The loss of chromosome integrity in *top2-5* is not a consequence of Cdc14-driven activities in anaphase.** A synchronous G1 release experiment was performed for isogenic *TOP2 cdc14-1* (left panels) and *top2-5 cdc14-1* (right panels) strains. (**a**) Charts depicting the cell cycle progression under the microscope. (**b**) FACS analysis of the DNA content. (**c**) EtBr staining of whole chromosomes resolved by PFGE. Note how chromosome behaviour of the *top2-5 cdc14-1* strain fully resembled that of *top2-5 cdc15-2*.

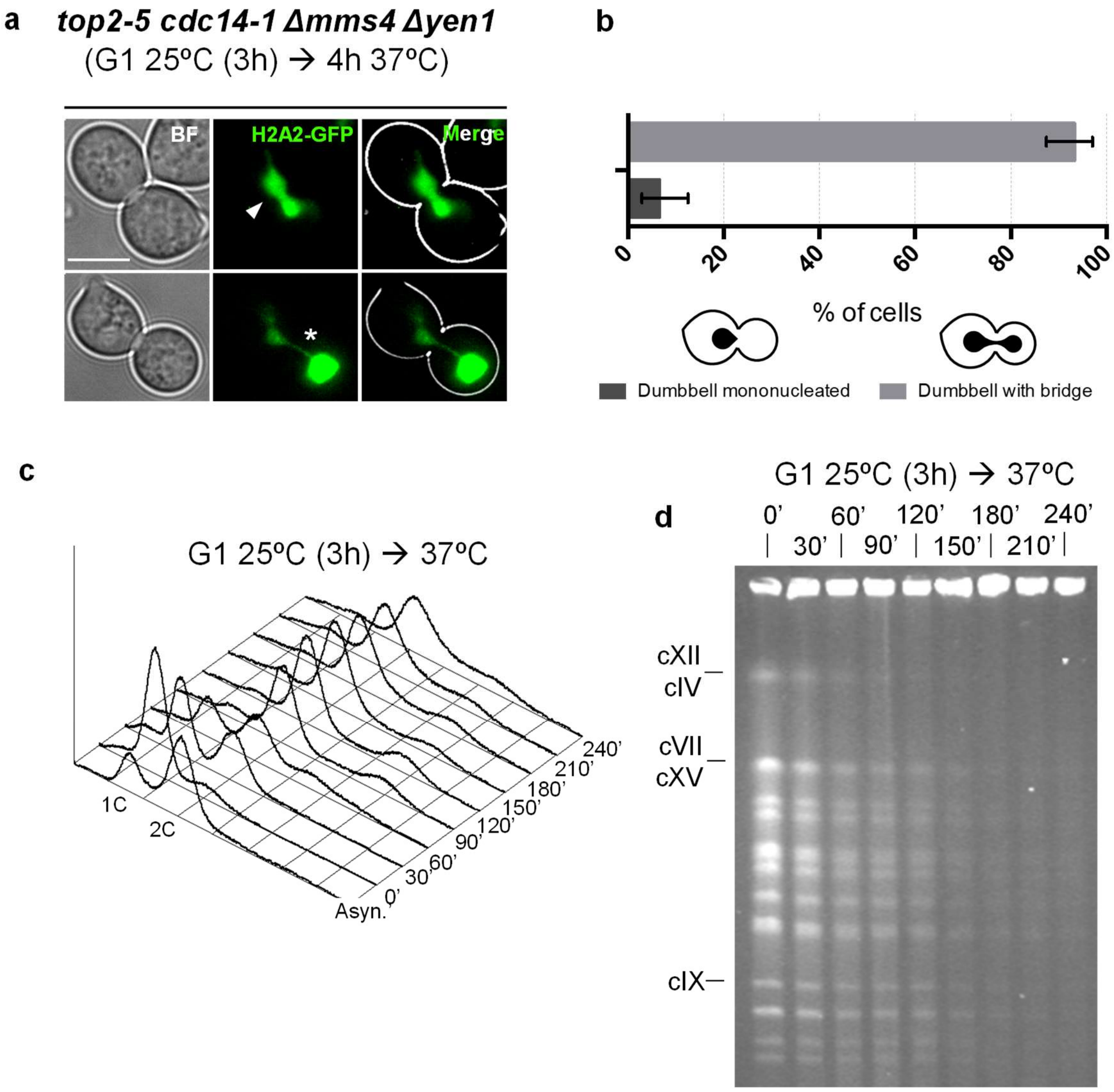


**Figure S9. The loss of chromosome integrity in *top2-5* occurs earlier in mutants for the major HR structure-specific endonucleases.** A synchronous G1 release experiment was performed for the *top2-5 cdc14*-1 Δ*mms4* Δ*yen1* strain. (**a**) Micrographs of representative cells 4 h after the G1 release. This strain resembles the previously characterized *top2-5 cdc14-1* anaphase bridge: a mixture of *top2-5 cdc15-2* bridges (arrow), and dumbbells with most of the histone signal in the daughter body (asterisk) [Ramos-Pérez et al. G3 (Bethesda). 7, 3379–3391 (2017)]. The scale bar depicts 5 µm. BF, bright field. (**b**) Chart of the proportion of the sum of these two phenotypes in the population. (**c**) FACS analysis of the DNA content. (**d**) EtBr staining of whole chromosomes resolved by PFGE. Note how the general chromosome shift starts taking place earlier than in other mutants and growth conditions (at around 60’).

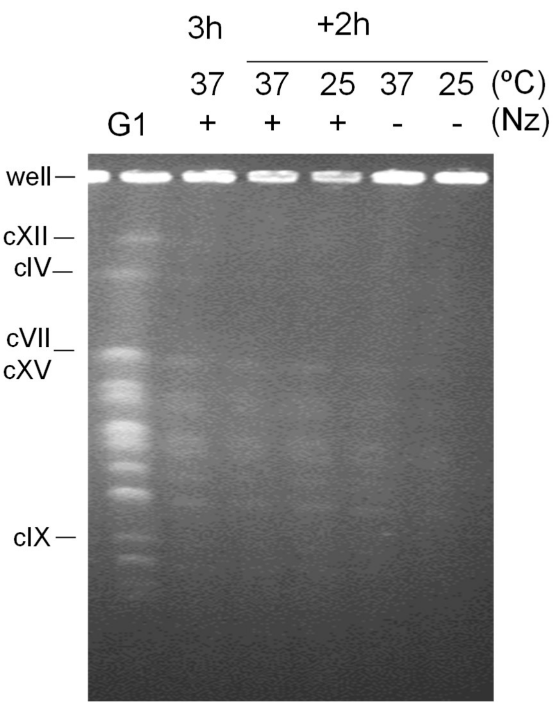


**Figure S10. The change in chromosome structure caused by the absence of Top2 is neither modified by previous treatment with Nocodazole nor restored by Top2 reactivation.** A synchronous G1 release experiment was performed for the *top2-5 cdc15-2* strain under restrictive conditions (37 ºC). At the time of the G1 release, Nocodazole (Nz) was added to depolymerize the spindle and transiently block cells in G2/M. After 3 h, the culture was split in four and incubated for another 2 h. One subculture was kept in Nz and at 37ºC; one kept the Nz but was shift back to 25 ºC (i.e., re-activate Top2); one was kept at 37 ºC while removing Nz; whereas the fourth one was treated to re-activate Top2 (25ºC shift) and remove the Nz. The PFGE of all intermediate and end point conditions is shown (the photo has been squeezed 1:2 vertically to fit in the figure). Note that chromosome structural shift was not reverted once established. This experiment relates to those in Figure 5.

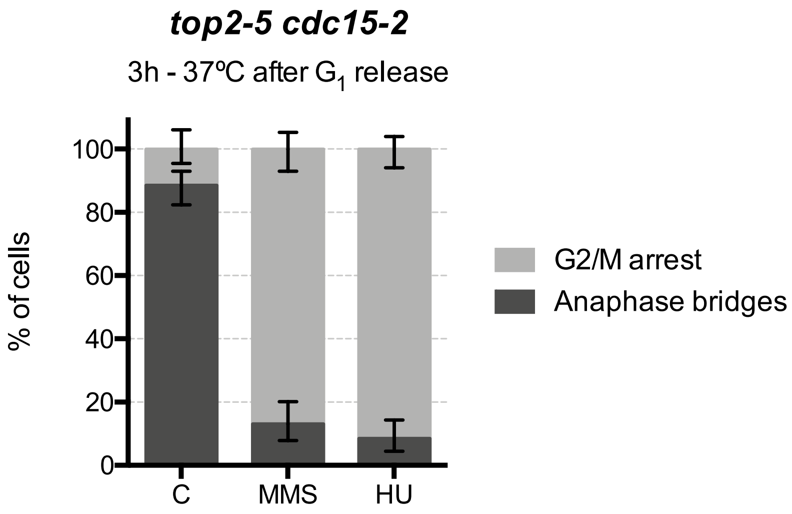


**Figure S11. The replication stress checkpoints are functional in the absence of Top2.** Synchronous G1 release experiments were performed for the *top2-5 cdc15-2* strain. At the time of the G1 release into 37 ºC, the culture was split in three. 2M Hydroxyurea (HU) was added to one subculture; 0.01 % w/v MMS to another one; and the third one was incubated without exogenous replication stress (C). Three hours after the G1 release, samples were observed under the microscope and classify as G2/M (mononucleated dumbbells) or with anaphase bridges (H2A2-GFP stretched across the dumbbell's neck). Error bars depict 95% confidence intervals.

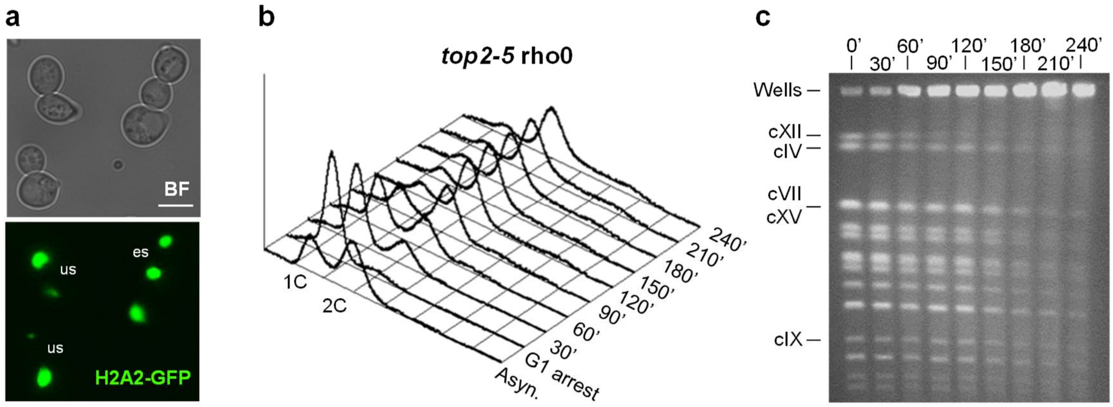


**Figure S12. Lack of mitochondrial function does not prevent the PFGE chromosome shift in *top2-5*.** A synchronous G1 release experiment was performed for a *top2-5 [rho0]* strain. (**a**) Micrograph of representative cells 4 h after the G1 release. Note the massive uneven segregation events of the H2A2-GFP signal, as in *top2-5* in Figure 1c; us, uneven/unequal segregation; es, even/equal segregation. (**b**) Flow cytometry (FACS) analysis of the DNA content. Note that, unlike *top2-5* in Figure 1d, the 2C peak remains at later time points, indicating a late anaphase/telophase arrest. (**c**) Ethidium bromide (EtBr) staining of whole chromosomes resolved by Pulsed-Field Gel Electrophoresis (PFGE). Note how the cXII in-gel signal is first to disappear and the other chromosome bands still get fainter after 150'.

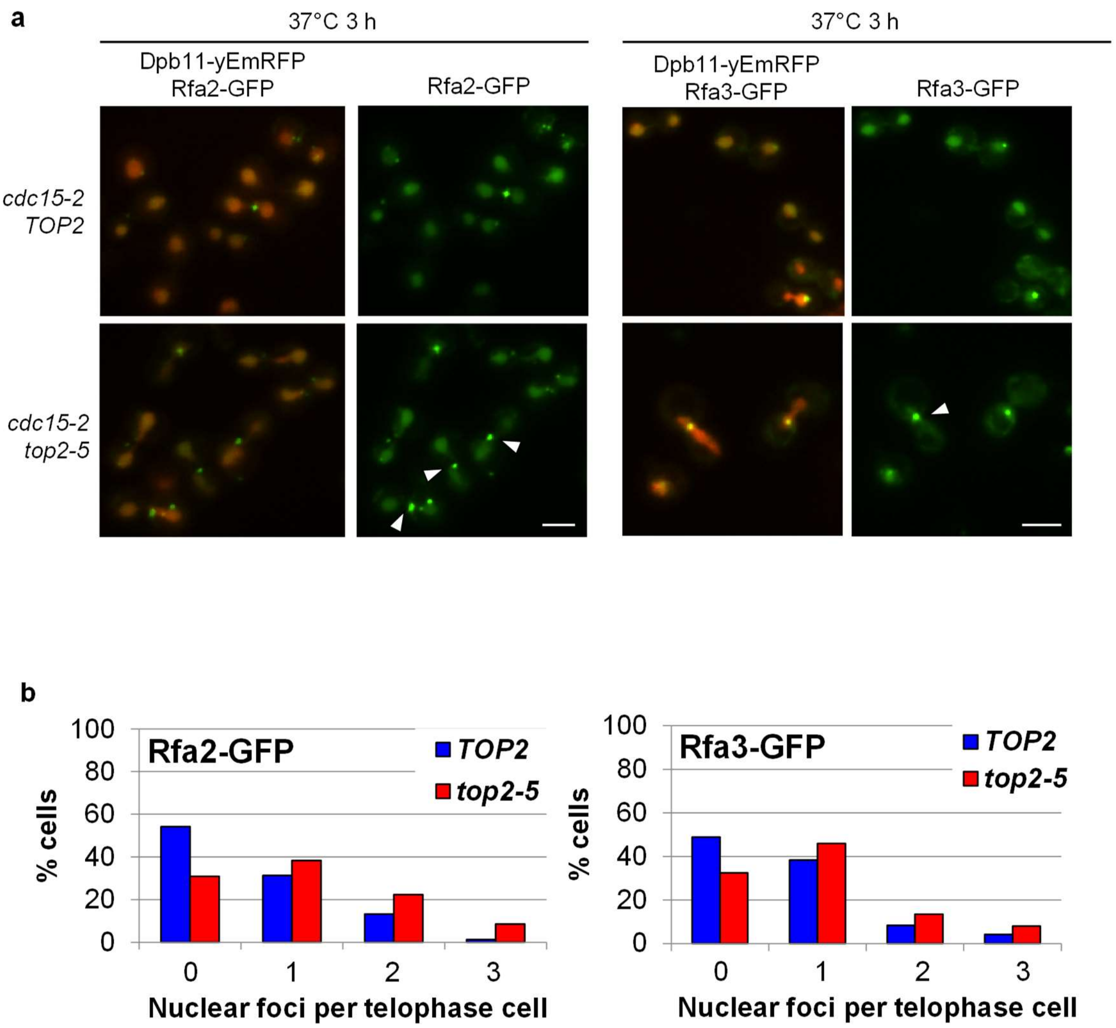


**Figure S13. There is more ssDNA in the *top2-5 cdc15-2* telophase block.** *TOP2* and *top2-5* strains bearing *cdc15-2*, the ultrafine anaphase bridge marker Dpb11-yEmRFP and one RPA component labelled with GFP (either Rfa2 or Rfa3) were blocked in telophase at 37 ºC. (**a**) Representative micrographs. Note how *top2-5* has more foci and they are more intense. Scale bars depict 5 μm. Arrowheads point to foci located at anaphase bridges. (**b**) Quantification of foci number per telophase cell (N>200). See also Table 1.

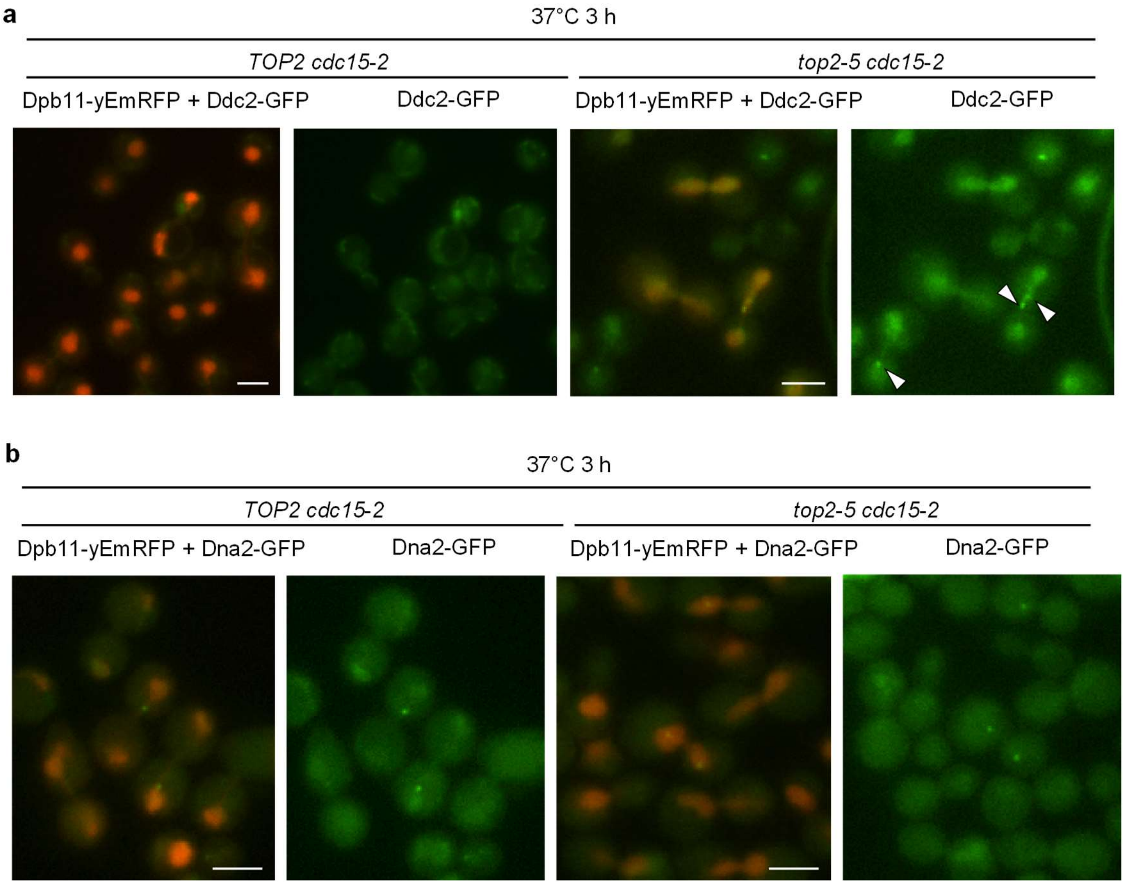


**Figure S14. The replication stress marker Ddc2 gets enriched in *top2-5 cdc15-2* telophase nuclei.** (**a**) Representative micrograph of the *TOP2 cdc15-2 DPB11-yEmRFP DDC2-GFP* and *top2-5 cdc15-2 DPB11-yEmRFP DDC2-GFP* strains blocked in in telophase at 37 ºC. Note that nuclear abundance of Ddc2 increases in *top2-5*, where it also forms foci. (**b**) Representative micrograph of the *TOP2 cdc15-2 DPB11-yEmRFP DNA2-GFP* and *top2-5 cdc15-2 DPB11-yEmRFP DNA2-GFP* strains blocked in in telophase at 37 ºC. Note that Dna2 forms foci in both telophase blocks, regardless the Top2 activity. Scale bars depict 5 µm. Arrowheads point to foci located at anaphase bridges. See also Table 1.

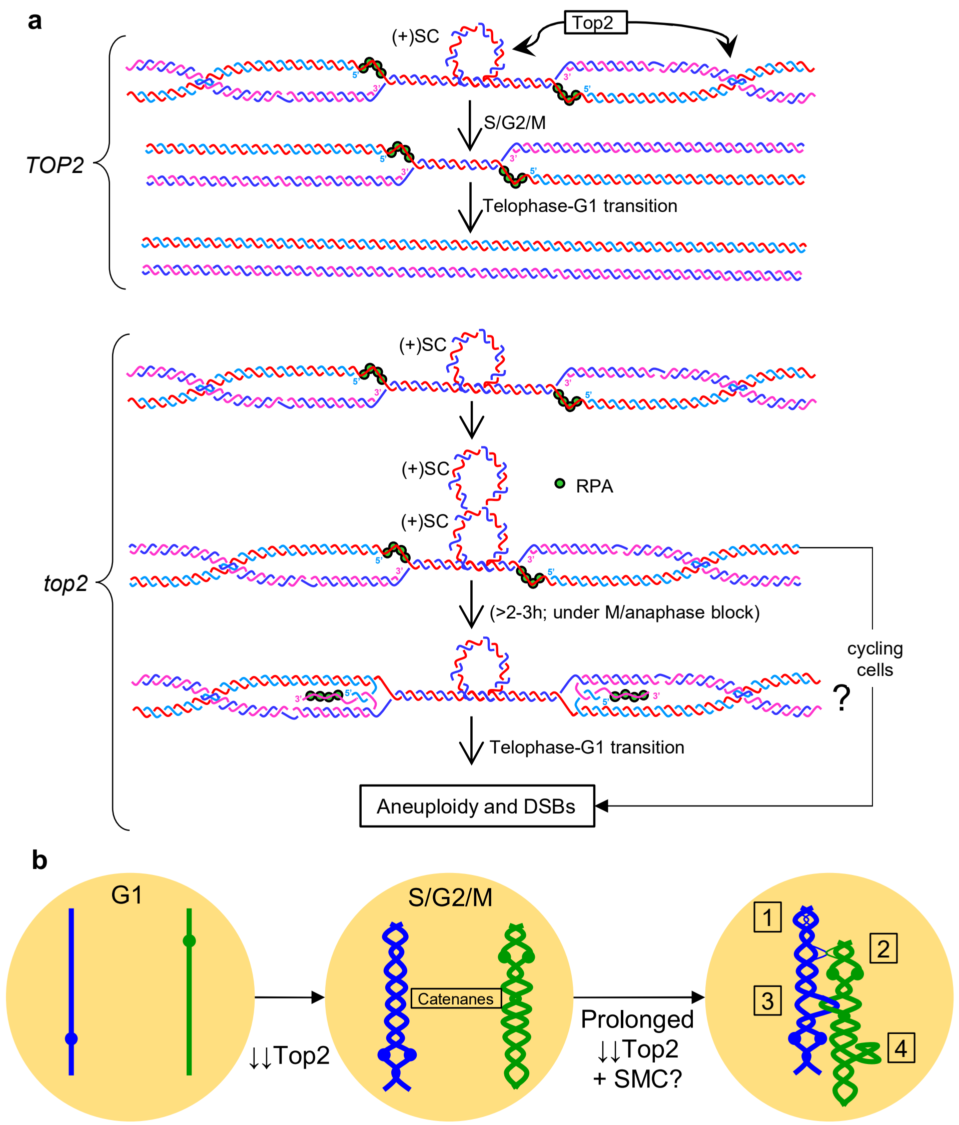


**Figure S15. Two putative models of how changes on the chromosome structure occur upon prolonged Top2 deficiency.** (**a**) When replication is ending and two converging forks are approaching, critical positive supercoiling arises ((+)SC) in between and precanenanes behind. Supercoiling interferes with replication completion, especially in difficult-to-replicate regions. Top2 deals with both topological problems, replication can finish, and sister chromatids are resolved for segregation. Part of these regions finish replication during the telophase-G1 transition, according to a recent report. In the prolonged absence of Top2, these regions accumulate an excess of topological problems that shift the chromosome structure so that chromosomes get trapped in a PFGE. Based on previous literature and the findings shown herein, we speculate that an excess of (+)SC may turn into reversed forks, and that these structures, or derivatives thereof, lead to PFGE entrapment. (**b**) Sister chromatid catenations arise during replication. Without Top2, this class of intertwining persists. Meanwhile, other DNA metabolic processes go on, such as transcription, DNA repair, cohesion and condensation, and these might exacerbate topological problems and other intertwining classes arise: 1, sister chromatid hemicatenanes; 2, chromosome hemicatenanes; 3, inter-chromosome knots; and 4, intra-chromosome knots. Unscheduled re-replication may also contribute to these problems. Both models are not mutually exclusive. RPA, replication protein A complex.

## SUPPLEMENTAL TABLES.

**Table S1. Strains used in this work.**

| Strain name | Relevant genotype[a] | Origin |
|---|---|---|
| CH326 | (S288C) *MAT***a** *ura3-52 his4-539am lys2-801am SUC2+ top2-5* | 1 |
| CH325 | (S288C) *MAT***a** *ura3-52 his4-539am lys2-801am SUC2+ top2-4* | 1 |
| CH335 | (S288C) *MAT***a** *ura3-52 his4-539am lys2-801am SUC2+ TOP2* | 1 |
| FM1386 | CH326; *H2A2(YBL003c):GFP:BleMX*; *Δbar1::URA3* | 2 |
| FM1387 | CH325; *H2A2(YBL003c):GFP:BleMX*; *Δbar1::URA3* | 2 |
| FM1419 | CH335; *H2A2(YBL003c):GFP:BleMX*; *Δbar1::URA3* | 2 |
| FM2021 | FM1386; *cdc15-2:9myc:Hph* | 2 |
| FM2086 | FM1419; *cdc15-2:9myc:Hph* | 2 |
| FM2154 | FM1386; *cdc14-1:9myc:Hph* | 2 |
| FM2152 | FM1419; *cdc14-1:9myc:Hph* | 2 |
| FM2170 | FM2154; *Δmms4::natMX4*; *Δyen1::KanMX4* | This work |
| FM2268 | FM2021; *Δrad52::KanMX4* | This work |
| FM2305 | FM1386 *[rho$^0$]* | This work[b] |
| E1670 | (W303) *MAT***a** *ade2-1 trp1-1 can1-100 his3-11,15 leu2- 3,112 RAD5 + GAL psi+ura3::URA3/GPD-TK$_{7x}$* | 3 |
| FM2128 | E1670; *cdc15-2:9myc:Hph* | This work[c] |
| FM2129 | E1670; *top2-5:NAT; cdc15-2:9myc:Hph* | This work[c] |
| FM2241 | *MAT**α** can1Δ::STE2pr-Sp_his5 lyp1Δ his3Δ1 leu2Δ0 ura3Δ0 met15Δ0 top2-5::natMX* | 2 |
| ML532 | *MAT**α** can1Δ::STE2pr-LEU2 lyp1Δ his3Δ1 leu2Δ0 ura3Δ0 TRP1 LYS2 MET15 KanMX:tTA(tetR-VP16)-tetO2-DPB11* | 4 |
| OQR84 | ML532; *KanMX:tTA(tetR-VP16)-tetO2-DPB11-yEmRFP; top2-5:NAT; cdc15-2:9myc:Hph* | This work[c,d] |
| OQR-TOP2-Xs-GFP (39 strains) | *MAT***a** *can1Δ::STE2pr-LEU2 lyp1Δ his3Δ1 leu2Δ0 ura3Δ0 MET15 KanMX:tTA(tetR-VP16)-tetO2-DPB11- yEmRFP; TOP2; cdc15-2:9myc:Hph; X-GFP:His3MX* | This work[e] |
| OQR-top2-5-Xs-GFP (39 strains) | *MAT***a** *can1Δ::STE2pr-LEU2 lyp1Δ his3Δ1 leu2Δ0 ura3Δ0 MET15 KanMX:tTA(tetR-VP16)-tetO2-DPB11- yEmRFP; top2-5:NAT; cdc15-2:9myc:Hph; X-GFP:His3MX* | This work[e] |

[a] Semicolons separate independent transformation events during strain construction. Intermediate strains are omitted.

[b] The mitochondrial DNA was eliminated by growing the strain under the presence of ethidium bromide as reported previously in [5].

[c] The ts alleles were transferred after PCR amplification from strains where they had been tagged downstream with the corresponding markers according to [6].

[d] The yEmRFP was tagged to DPB11 through an adaptamer-mediated PCR fusion methodology described in [7].

[e] These strains were selected after crossing OQR84 with the GFP tagged collection reported in [8], whose basic genotype is *MAT***a** *his3Δ1 leu2Δ0 met15Δ0 ura3Δ0 X-GFP(S65T):His3MX* (*X* being the gene of interest). Individual genotypes are omitted for the sake of space. The haploids were selected on minimum media plates supplemented with canavanine, thialysine, G418 and hygromycin, and in the absence of histidine, leucine and methionine. Then, the presence of either *TOP2* or *top2-5:NAT* was checked by nourseothricin sensitivity or resistance, respectively.

## SUPPLEMENTAL REFERENCES

1. Holm, C., Goto, T., Wang, J. C. & Botstein, D. DNA topoisomerase II is required at the time of mitosis in yeast. *Cell* **41**, 553–63 (1985).

2. Ramos-Pérez, C. *et al.* Genome-Scale Genetic Interactions and Cell Imaging Confirm Cytokinesis as Deleterious to Transient Topoisomerase II Deficiency in Saccharomyces cerevisiae. *G3 (Bethesda).* **7**, 3379–3391 (2017).

3. Lengronne, A., Pasero, P., Bensimon, A. & Schwob, E. Monitoring S phase progression globally and locally using BrdU incorporation in TK(+) yeast strains. *Nucleic Acids Res.* **29**, 1433–42 (2001).

4. Germann, S. M. *et al.* Dpb11/TopBP1 plays distinct roles in DNA replication, checkpoint response and homologous recombination. *DNA Repair (Amst).* **10**, 210–24 (2011).

5. Anaissi-Afonso, L. *et al.* Lawsone, Juglone, and β-Lapachone Derivatives with Enhanced Mitochondrial-Based Toxicity. *ACS Chem. Biol.* **13**, 1950–1957 (2018).

6. Smith, J. S. & Burke, D. J. *Yeast Genetics: Methods and Protocols*. **1205**, (Springer New York, 2014).

7. Reid, R. J. D., Lisby, M. & Rothstein, R. Cloning-free genome alterations in Saccharomyces cerevisiae using adaptamer-mediated PCR. *Methods Enzymol.* **350**, 258–77 (2002).

8. Huh, W. K. *et al.* Global analysis of protein localization in budding yeast. *Nature* **425**, 686–691 (2003).
